# Immunological effects of adjuvants in subsets of antigen presenting cells of cancer patients undergoing chemotherapy

**DOI:** 10.1186/s12967-020-02218-x

**Published:** 2020-01-23

**Authors:** Angela Mauriello, Carmen Manolio, Beatrice Cavalluzzo, Antonio Avallone, Marco Borrelli, Alessandro Morabito, Emanuele Iovine, Angela Chambery, Rosita Russo, Maria Lina Tornesello, Franco M. Buonaguro, Maria Tagliamonte, Luigi Buonaguro

**Affiliations:** 1 Laboratory of Cancer Immunoregulation, Istituto Nazionale per lo Studio e la Cura dei Tumori, IRCCS “Fondazione Pascale”, Via Mariano Semmola, 80131 Naples, Italy; 2 GI Medical Oncology, Istituto Nazionale per lo Studio e la Cura dei Tumori, IRCCS “Fondazione Pascale”, Naples, Italy; 3 Thoracic Medical Oncology, Istituto Nazionale per lo Studio e la Cura dei Tumori, IRCCS “Fondazione Pascale”, Naples, Italy; 4 Environmental, Biological and Pharmaceutical Science and Technology Dept, Università della Campania “Luigi Vanvitelli”, Caserta, Italy; 5 Laboratory of Molecular Biology and Viral Oncology, Istituto Nazionale per lo Studio e la Cura dei Tumori, IRCCS “Fondazione Pascale”, Naples, Italy

**Keywords:** Adjuvant, Colon cancer, Lung cancer, Immune-response, Cancer vaccine

## Abstract

**Background:**

We have previously shown that HCC patients and healthy subjects are equally responsive to a RNAdjuvant^®^, a novel TLR-7/8/RIG-I agonist based on noncoding RNA developed by CureVac, by an ex vivo evaluation. However, the immunological effect of adjuvants on immune cells from cancer patients undergoing chemotherapy remains to be demonstrated. Different adjuvants currently used in cancer vaccine clinical trials were evaluated in the present study on immune cells from cancer patients before and after chemotherapy in an ex vivo setting.

**Methods:**

PBMCs were obtained from 4 healthy volunteers and 23 patients affected by either colon (OMA) or lung cancer (OT). The effect of CpG, Poly I:C, Imiquimod and RNA-based adjuvant (RNAdjuvant^®^) was assessed using a multiparametric approach to analyze network dynamics of early immune responses. Evaluation of CD80, CD86 and HLA-DR expression as well as the downstream effect on CD4^+^ T cell phenotyping was performed by flow cytometry; cytokine and chemokine production was evaluated by Bio-Plex ProTM.

**Results:**

Treatment with RNAdjuvant^®^ induced the strongest response in cancer patients in terms of activation of innate and adoptive immunity. Indeed, CD80, CD86 and HLA-DR expression was found upregulated in circulating dendritic cells, which promoted a CD4^+^ T cell differentiation towards an effector phenotype. RNAdjuvant^®^ was the only one to induce most of the cytokines/chemokines tested with a pronounced Th1 cytokine pattern. According to the different parameters evaluated in the study, no clear cut difference in immune response to adjuvants was observed between healthy subjects and cancer patients. Moreover, in the latter group, the chemotherapy treatment did not consistently correlate to a significant altered response in the different parameters.

**Conclusions:**

The present study is the first analysis of immunological effects induced by adjuvants in cancer patients who undergo chemotherapy, who are enrolled in the currently ongoing cancer vaccine clinical trials. The results show that the RNAdjuvant^®^ is a potent and Th1 driving adjuvant, compared to those tested in the present study. Most importantly, it is demonstrated that chemotherapy does not significantly impair the immune system, implying that cancer patients are likely to respond to a cancer vaccine even after a chemotherapy treatment.

## Background

Cancer immunotherapy is a recently fast growing field and several therapeutic cancer vaccine strategies are being investigated [1]. However, the efficacy of such strategies is often limited, due to the low immunogenicity of administered antigens which requires to be potentiated by a strong adjuvant [2–4]. Although the field is continuously evolving, only a few number of adjuvants have been approved for human use to date [5].

The development of new adjuvants has been guided in the last years by the detailed comprehension of the central role played by the innate immunity to initiate and direct the adaptive immune response. In particular, the pattern recognition receptors (PRRs) in cells of the innate immune system act as sensors for bacterial and viral nucleic acids (pathogen-associated molecular patterns; PAMPs) and induce the innate immune response by activation of a signaling cascade resulting in the upregulation of inflammatory cytokines, chemokines, and type I IFNs. This will eventually end up in triggering a robust adaptive immune response [6]. Therefore, PRRs represent the ideal target for vaccine adjuvants to initiate and boost an antigen-specific T cell response (reviewed in [7]).

In this framework, several TLR ligands have been extensively evaluated as vaccine adjuvants in pre-clinical as well as human clinical setting (reviewed in [8]). In particular, the polyinosinic–polycytidylic acid (Poly I:C) is a synthetic TLR-3 agonist reported as type 1 adjuvant able to elicit antigen-specific CTL, antibody and Th1 type immune responses when included in cancer vaccines [9–11]. Synthetic CpG oligodeoxynucleotides (ODNs) are TLR-9 agonists able to induce type I IFNs as well as proinflammatory cytokine production, generating Th1 type cellular and humoral immune responses [12, 13]. Recently, the CpG-ODN was approved for the first time for application in humans, and Heplisav-B, a hepatitis B vaccine containing CpG-ODN as an adjuvant, was approved by the United States Food and Drug Administration [14]. Imiquimod, is a synthetic TLR7/8 agonist, reported to induce an adjuvant activity able to boost antigen-specific humoral and Th1 type cellular immune responses [15, 16]. However, systemic toxicity has been reported and the only TLR7 agonist-based drug approved for clinical use as an anti-tumor agent is the topically applied Aldara cream for patients with precancerous skin lesions [17]. Nevertheless, the field is still open for new adjuvants and in particular for molecules able to elicit a balanced humoral and cellular immune response for potential application in both preventive and therapeutic vaccines.

In this framework, RNAdjuvant^®^, a TLR 7/8/RIG I agonist based on noncoding RNA has been shown to induce a potent Th1 and long-lasting immune response if used as adjuvant for peptide vaccines in preclinical models, including cancer tumor models [18, 19].

Therapeutic cancer vaccines are designed to be administered in patients already affected by a disease such as cancer which is considered to induce an immune compromised situation, especially as consequence of standard-of-care chemotherapy. Indeed, the cytostastic effect is not specific to cancer cells and may affect all replicating cells, including immune cells, possibly resulting in the impairment of effector T cells and activation of immunosuppressive mechanisms [20]. However, the lymphodepletion caused by chemotherapy can turn into a reset of the immune system as consequence of the rebound replenishment of immune cells [21], with the emergence of competent effector cells also with anticancer activity [22]. Indeed, cancer patients have been shown to respond to influenza virus vaccination during chemotherapy [23, 24] and specific anti-infective vaccination guidelines for patients with hematological malignancies have been designed [25].

These observations indicate that routine chemotherapy is compatible with the initiation of an immune response and anticancer vaccines have been shown to elicit anti-tumor immune responses in patients during chemotherapy (reviewed in [26]).

Adjuvants are a key component of vaccine formulations, historically used as preventive strategy in healthy subjects. Provided all the immunological implications related to the cancer disease and the chemotherapy, an experimental validation is needed to prove that adjuvants are equally effective in healthy subjects and cancer patients. For such a reason, taking advantage of an ex vivo multiparametric platform, developed and fully validated by our group [27–33], we have previously shown that HCC patients who do not undergo chemotherapy and healthy subjects are equally responsive to the RNAdjuvant^®^ [34]. However, the immunological effect of adjuvants in cancer patients undergoing chemotherapy remains to be demonstrated.

To this aim, adjuvants currently used in cancer vaccine clinical trials were evaluated in an ex vivo setting on immune cells from patients affected by colon cancer and lung cancer before and after chemotherapy.

Among the adjuvants evaluated in the study, the RNAdjuvant^®^ elicited the strongest response in cancer patients in terms of activation of innate and adoptive immunity. It was the only one to induce production of IFNγ Th1 cytokine and a potent inducer of both pro-inflammatory cytokines and chemokines. Very importantly, immune response induced in cancer patients was significant although of lower potency compared to healthy subjects. Nevertheless, the narrow differences between pre and post-chemotherapy samples indicates that cancer patients may well respond to therapeutic cancer vaccines even after chemotherapy.

## Materials and methods

### Enrolled subjects

Peripheral blood was obtained by venipuncture from 4 healthy volunteers, eleven colon cancer (OMA) and twelve lung cancer patients (OT) before and after chemotherapy. Post-chemotherapy blood samples were obtained about 3 weeks after the end of therapy (median 25 days). All human specimens were obtained and processed at the National Cancer Institute in Naples under informed consent, as approved by the Institutional Review Board.

### PBMC isolation

Fresh human PBMCs were isolated by Ficoll-Hypaque density gradient centrifugation and plated in six-well plates at a concentration of approximately 1 × 10^6^ cells/well in a maximum volume of 2 ml/well. Isolated PBMCs were incubated for 24 h (short-term culture) or for 7 days (medium-term culture) in RPMI 1640 medium.

### Cell culture medium

PBMCs culture medium consisted of RPMI 1640 medium (Life Technologies, Carlsbad, CA) supplemented with 2 mM l-glutamine (Sigma), 10% fetal calf serum (Life Technologies) and 2% penicillin/streptomycin (5000 I.U./5 mg/ml, MP Biomedicals). Recombinant interleukin-2 (rIL-2; R&D Systems, Minneapolis, Minn.) was added at a concentration of 75 U/ml for medium-term culture (every 2 days).

### Cell treatment

PBMCs were incubated for 24 h (short-term culture) with the different adjuvants: 20 μg/ml of RNAdjuvant^®^ provided by CureVac (Tübingen, Germany), 6 μg/ml of Poly I:C, 5 μg/ml of Imiquimod, 2,5 μg/ml of CpG ODN 2006, 2 μg/ml of lipopolysaccharide (LPS) as positive control (from *Salmonella enterica serotype Minnesota*, purity > 99.0%). Alternatively, PBMCs were incubated for 7 days (medium-term culture) with the same concentration of RNAdjuvant^®^ or LPS, plus IL-2 added at day 0, 2 and 4. PBS was used as negative control. At the end of the incubation, PBMCs were harvested, washed with PBS 1× (137 mM NaCl, 2.7 mM KCl, 10 mM Na_2_HPO4, 2 mM KH_2_PO_4_, pH 7.2) without Calcium and Magnesium and analyzed by flow cytometry. All cell supernatants were collected for quantification of cytokine and chemokine production.

### Flow cytometry

Short-term culture PBMCs were incubated for 30 min at 4 °C with human monoclonal antibodies specific for CD80, CD86, HLA-DR, CD123, CD11c and CD14 (BD Pharmingen, San Diego, CA), washed and then analyzed with the Attune NxT flow cytometer (Life Technology). In particular, monocytes, mDCs and pDCs were identified according to the following gating strategy. The monocyte fraction was selected from the whole PBMCs and, within such a fraction, cells were selected according to the positivity for CD14 marker (CD14^+^: monocytes; CD14^−^: dendritic cells). CD14^−^ DCs were further divided in mDCs, if positive for CD11c, and pDCs, if positive for CD123. On cell subtypes defined in this way, the percentage of CD80, CD86 and HLA-DR positivity was assessed.

### T-maturation analysis

Medium-term culture PBMCs were analyzed for markers of CD4^+^ Tcell phenotyping by flow cytometry. PBMCs were collected, washed in PBS, centrifuged and incubated for 30 min. at 4 °C with monoclonal antibodies specific for the surface molecules of human T lymphocytes: CD4, CD45RA, CD45RO, CD62L (BD Pharmingen, San Diego, CA). Naïve (TN) CD4^+^ T were identified as CD4^+^/CD45RA^+^/CD62L^+^; effector cells (TE) as CD4^+^/CD45RA^+^/CD62L^−^; central memory (TCM) as CD4^+^/CD45RO^+^/CD62L^+^; effector memory (TEM) as CD4^+^/CD45RO^+^/CD62L^−^.

### Cytokine analysis

At the time of cell harvest, supernatants were also collected and stored at − 80 °C until use. Cytokine and chemokine production was evaluated by using a Bio-Plex MAGPIX Multiplex Reader system (Bio-Rad, Milan, Italy) equipped with a Bio-Plex Manager software v 6.1 (BioRad) according to manufacturer’s instructions [35]. All washing steps were performed on the Bio-Plex magnetic wash station (BioRad). Measurements were performed in triplicate on samples (50 µl) using the Bio-Plex Pro Human Cytokine 40-plex assay kit (Cat. No. 171AK99MR2, BioRad). Standard curves optimization and the calculation of analyte concentrations were performed by using the Bio-Plex Manager software. Data were expressed as mean ± SD.

### Statistical analysis

Statistical analysis was performed using Graphpad Prism 6 software (Graphpad Software, La Jolla, CA, USA) and the results of ANOVA nonparametric test were considered statistically significant at a level of p < 0.05. Normally distributed data were represented as mean ± S.E.M.

## Results

### Clinical parameters of subjects included in the analysis

Twenty-three subjects were enrolled in the present study. Eleven subjects were affected by colon cancer (OMA); twelve subjects were affected by non-small cell lung carcinoma (OT). Fourteen were males and nine were females with a median age of 65 (range 52–81). Four healthy donors were enrolled as controls. Clinical parameters and chemotherapy treatment of enrolled subjects are described in Table 1. Due to technical reasons, only three samples from OT patients were included in the present analysis.

**Table 1:** Informations and chemotherapy treatment of enrolled patients

Samples	Sex	Age	Treatment
Healthy 1	M	50	
Healthy 2	F	40	
Healthy 3	M	28	
Healthy 4	F	23	
OT 1	M	72	Cisplatin + vinorelbine
OT 2	F	73	Gemcitabine
OT 3	M	72	Carboplatin + gemcitabine
OT 4	F	68	Cisplatin + etoposide
OT 5	F	81	Carboplatin + alimta
OT 6	M	61	Cisplatin + gemcitabine
OT 7	M	62	Carboplatin + gemcitabine
OT 8	F	69	Cisplatin + gemcitabine
OT 9	M	72	Carboplatin + vinorelbine
OT 10	M	78	Cisplatin + gemcitabine
OT 11	F	64	Carboplatin + pemetrexed
OT 12	F	62	Cisplatin + pemetrexed
OMA 1	F	61	XELOX
OMA 2	F	54	XELOX
OMA 3	M	56	XELOX
OMA 4	M	62	XELOX
OMA 5	M	52	XELOX
OMA 6	F	60	XELOX
OMA 7	M	62	XELOX
OMA 8	M	60	XELOX
OMA 9	M	66	XELOX
OMA 10	M	55	XELOX
OMA 11	M	66	XELOX

### Basal expression of maturation markers in circulating APCs

The expression of CD80, CD86 and HLA-DR molecules was examined in circulating antigen-presenting cells (APCs) by flow cytometry. In particular, their expression on CD14^+^ monocytes, CD14^−^CD11c^+^ myeloid dendritic cells (mDCs) and CD14^−^CD123^+^ plasmacytoid dendritic cells (pDCs) were evaluated in parallel [34].

**Fig. 1:**
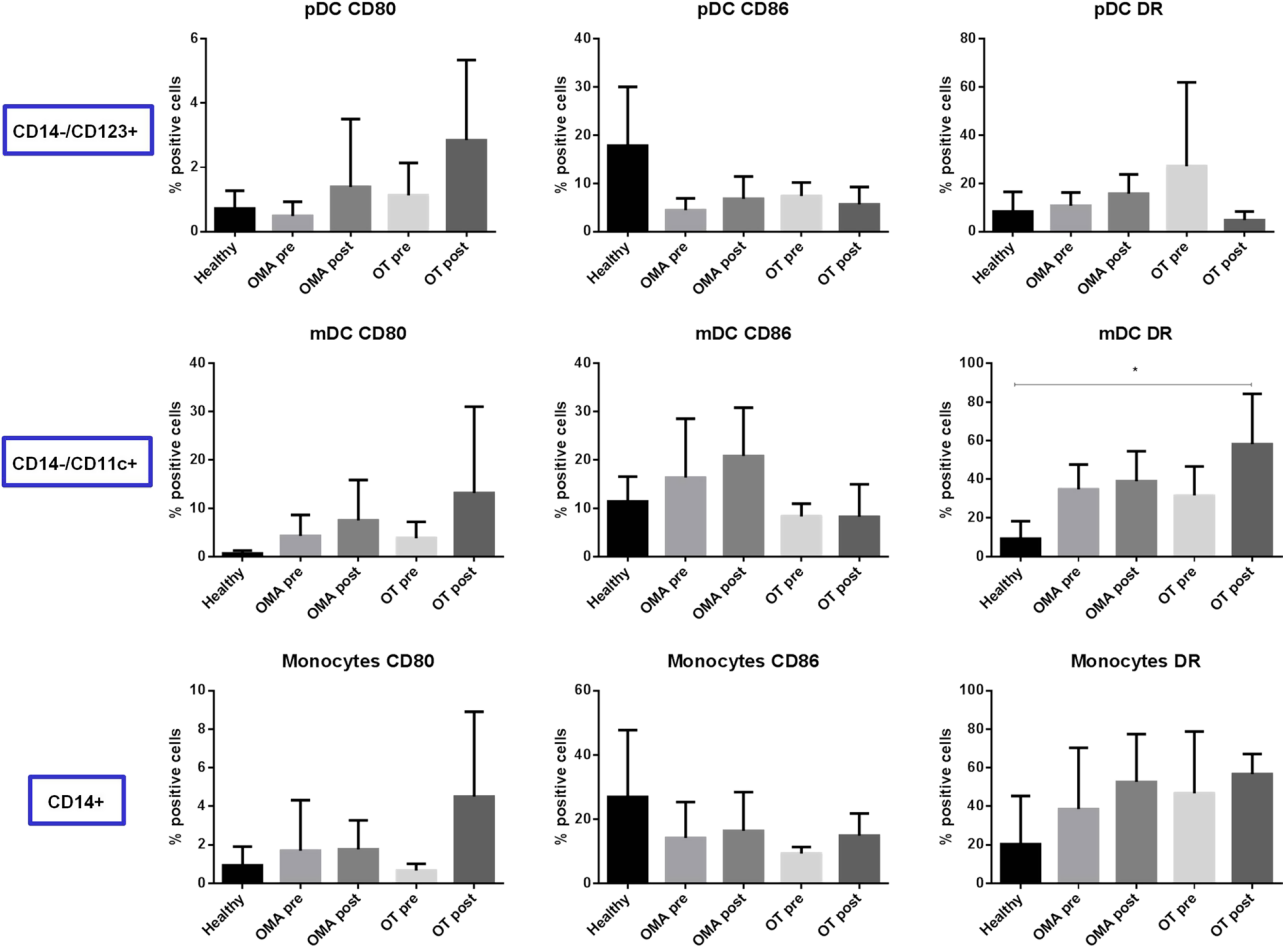
Basal expression of markers in circulating APCs. Each of the indicated markers was evaluated by flow cytometry on cells at basal level. Results are expressed of percentage of positive cells in the analyzed samples

The basal expression of the CD80, CD86 and HLA-DR molecules in the three APC populations was largely comparable between the healthy subjects and the cancer patients, before and after chemotherapy (Fig. 1). In particular, CD80 and HLA-DR were very low in healthy subjects and showed a trend of increased expression in cancer patients, especially after chemotherapy, with different degree in the three cell populations. Such an increased expression reached the statistical significance only for HLA-DR in monocytes from OT patients after chemotherapy. On the contrary, CD86 expression was high in healthy subjects and showed limited variation in cancer patients, without reaching a statistical significance (Fig. 1).

### Induction of maturation markers in plasmacytoid dendritic cells by different adjuvants

**Fig. 2:**
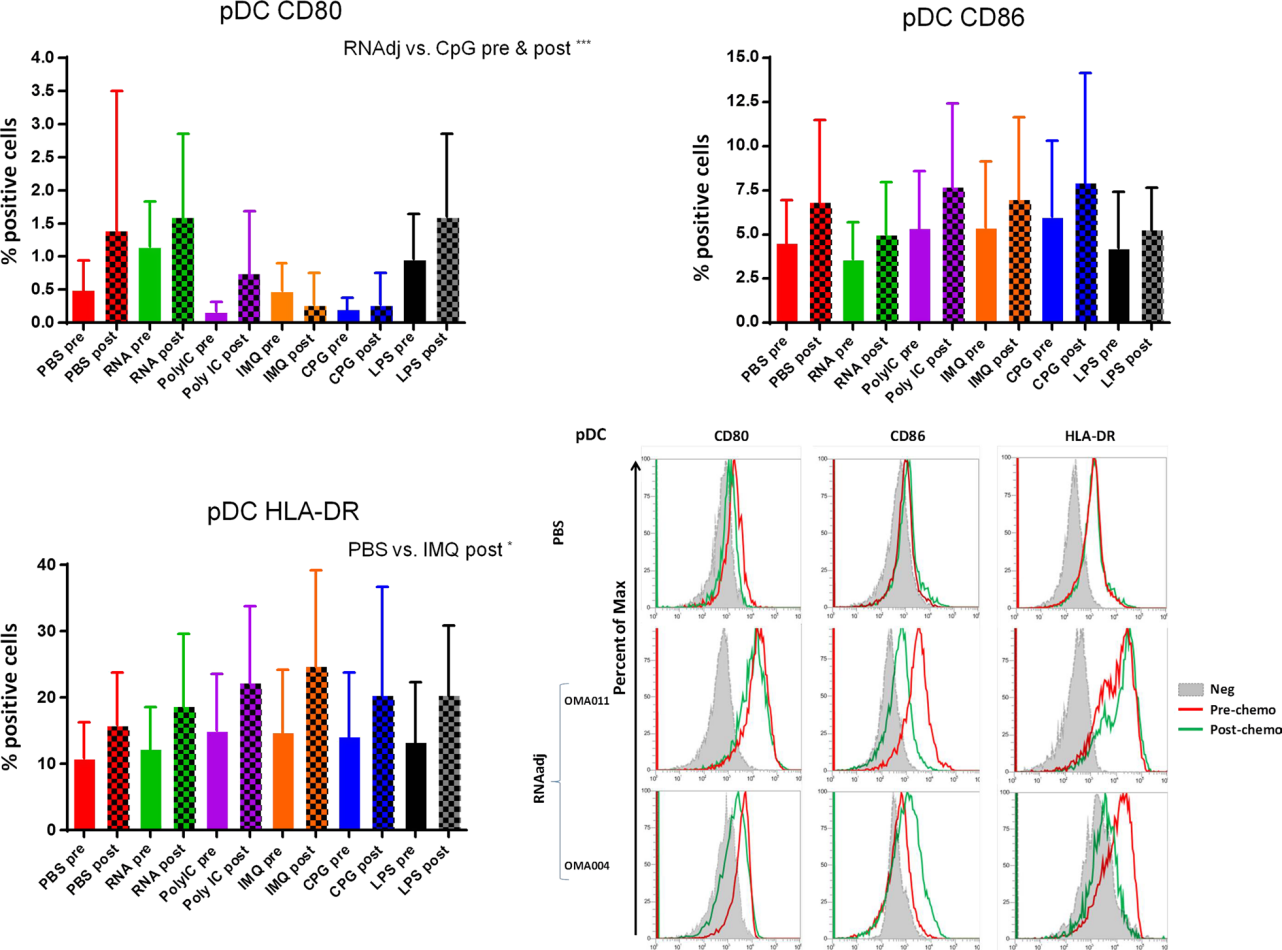
Expression of markers induced by adjuvants in circulating pDCs from colon ca patients. Each of the indicated markers was evaluated by flow cytometry on cells after ex vivo treatment with adjuvants. Samples from colon cancer patients were collected pre and post-chemotherapy. Results are expressed of percentage of positive cells in the analyzed samples. Expression of individual markers induced by RNAdjuvant^®^ in two individual patients is shown as plot

**Fig. 3:**
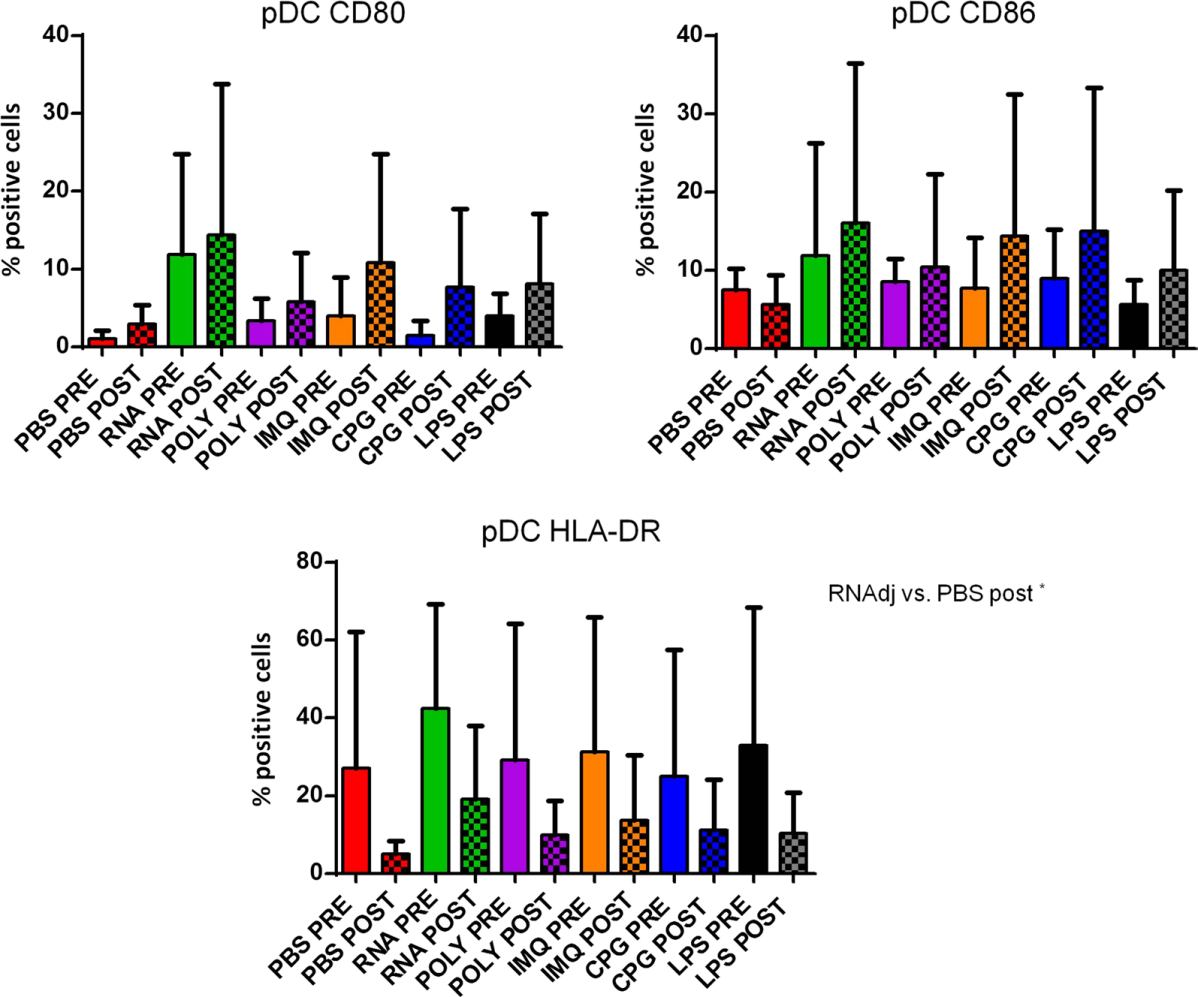
Expression of markers induced by adjuvants in circulating pDCs from lung ca patients. Each of the indicated markers was evaluated by flow cytometry on cells after ex vivo treatment with adjuvants. Samples from lung cancer patients were collected pre and post-chemotherapy. Results are expressed of percentage of positive cells in the analyzed samples

**Fig. 4:**
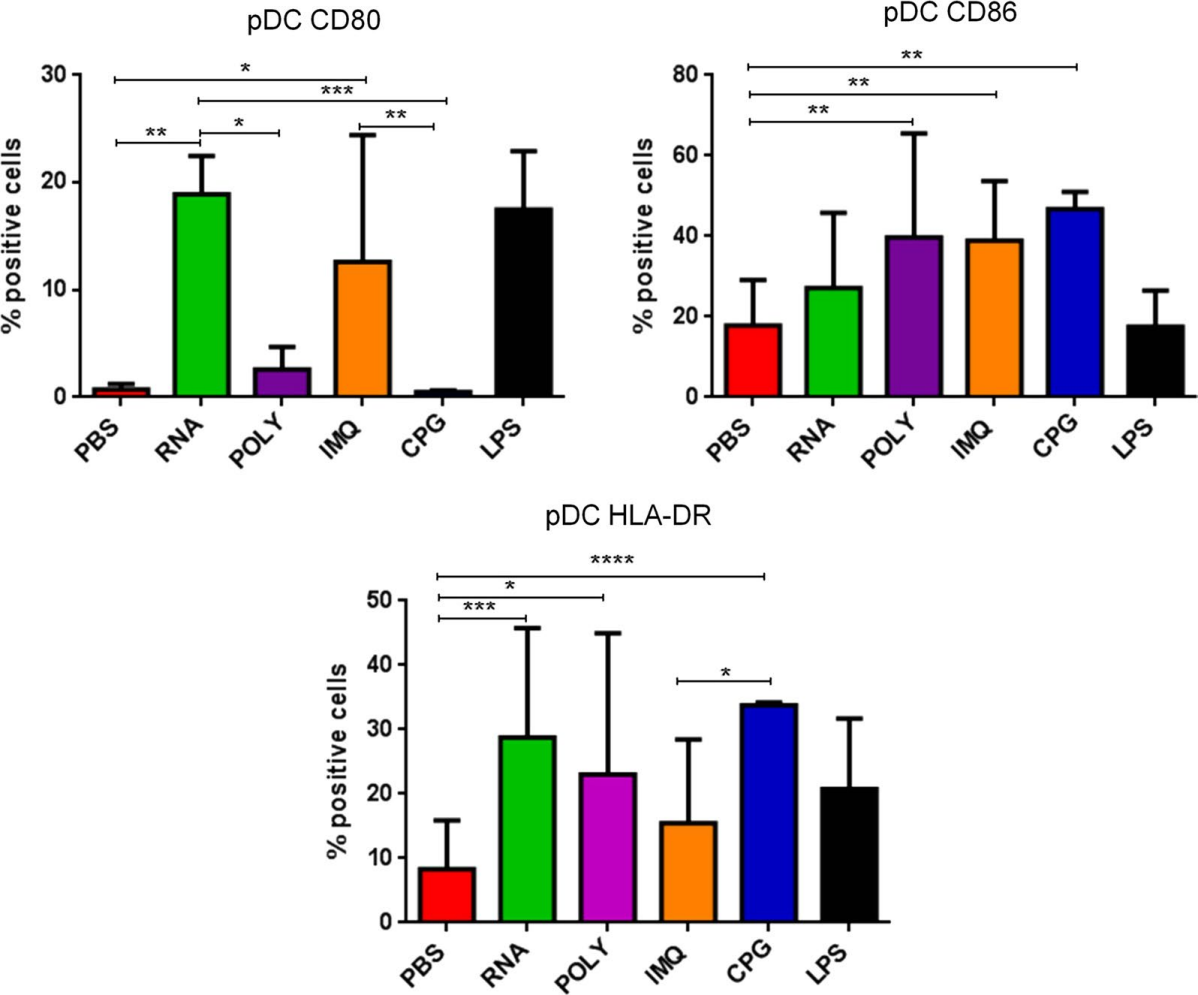
Expression of markers induced by adjuvants in circulating pDCs from healthy subjects. Each of the indicated markers was evaluated by flow cytometry on cells after ex vivo treatment with adjuvants. Results are expressed of percentage of positive cells in the analyzed samples

The expression of CD80, CD86 and HLA-DR molecules was evaluated in CD14^−^CD123^+^ plasmacytoid dendritic cells (pDCs) from cancer patients, before and after chemotherapy, upon ex vivo treatment with the adjuvants. The dose used for each adjuvant was selected according to recommendations provided by producers and reported in the literature [19, 34, 36–38]. Results in OMA patients showed that none of the adjuvants used in this study was able to induce a statistical significant increased expression in pDCs of the maturation markers compared to the negative control. The only exception was the IMQ that induced an upregulation of HLA-DR molecules in pDCs after the chemotherapy (Fig. 2; Additional file 1: Figure S1). Patients showed a great variable responsiveness to adjuvants and an example of low and high responders is shown in Fig. 2. Similar results were observed in pDCs derived from OT patients. In this setting, the RNAdjuvant^®^ was the only ex vivo treatment to induce a trend of increased expression of CD80 and HLA-DR compared to the negative control, in pre and post-treatment samples respectively (Fig. 3; Additional file 1: Figure S2). In healthy subjects the effects of adjuvants were evident and statistically significant. In particular, the expression of the three activation markers was increased by the adjuvants compared to negative control, with individual specificity. The comparison between the different adjuvants showed unique features in the induction of individual activation markers (Fig. 4).

### Induction of maturation markers in myeloid dendritic cells by different adjuvants

**Fig. 5:**
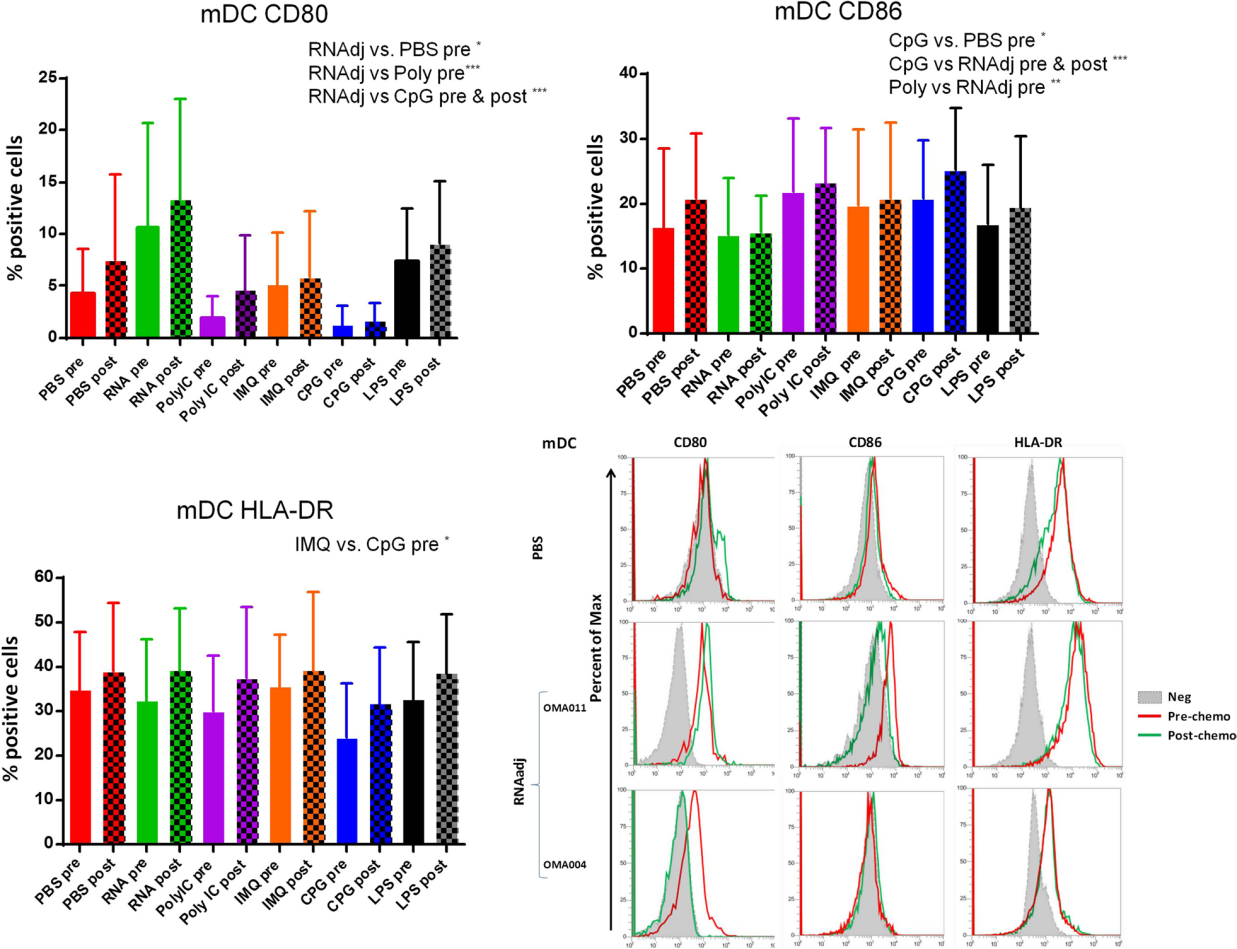
Expression of markers induced by adjuvants in circulating mDCs from colon ca patients. Each of the indicated markers was evaluated by flow cytometry on cells after ex vivo treatment with adjuvants. Samples from colon cancer patients were collected pre and post-chemotherapy. Results are expressed of percentage of positive cells in the analyzed samples. Expression of individual markers induced by RNAdjuvant^®^ in two individual patients is shown as plot

**Fig. 6:**
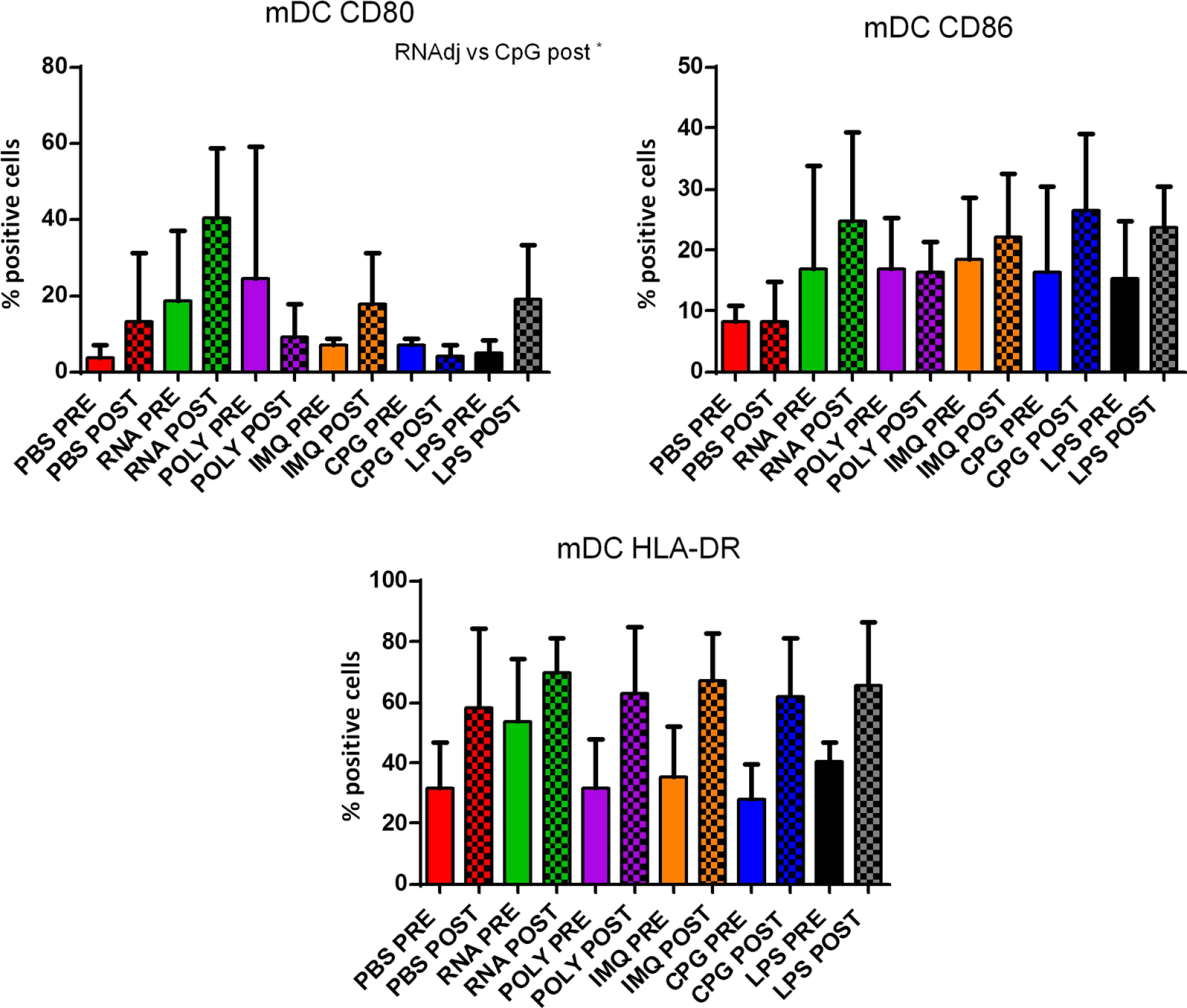
Expression of markers induced by adjuvants in circulating mDCs from lung ca patients. Each of the indicated markers was evaluated by flow cytometry on cells after ex vivo treatment with adjuvants. Samples from lung cancer patients were collected pre and post-chemotherapy. Results are expressed of percentage of positive cells in the analyzed samples

**Fig. 7:**
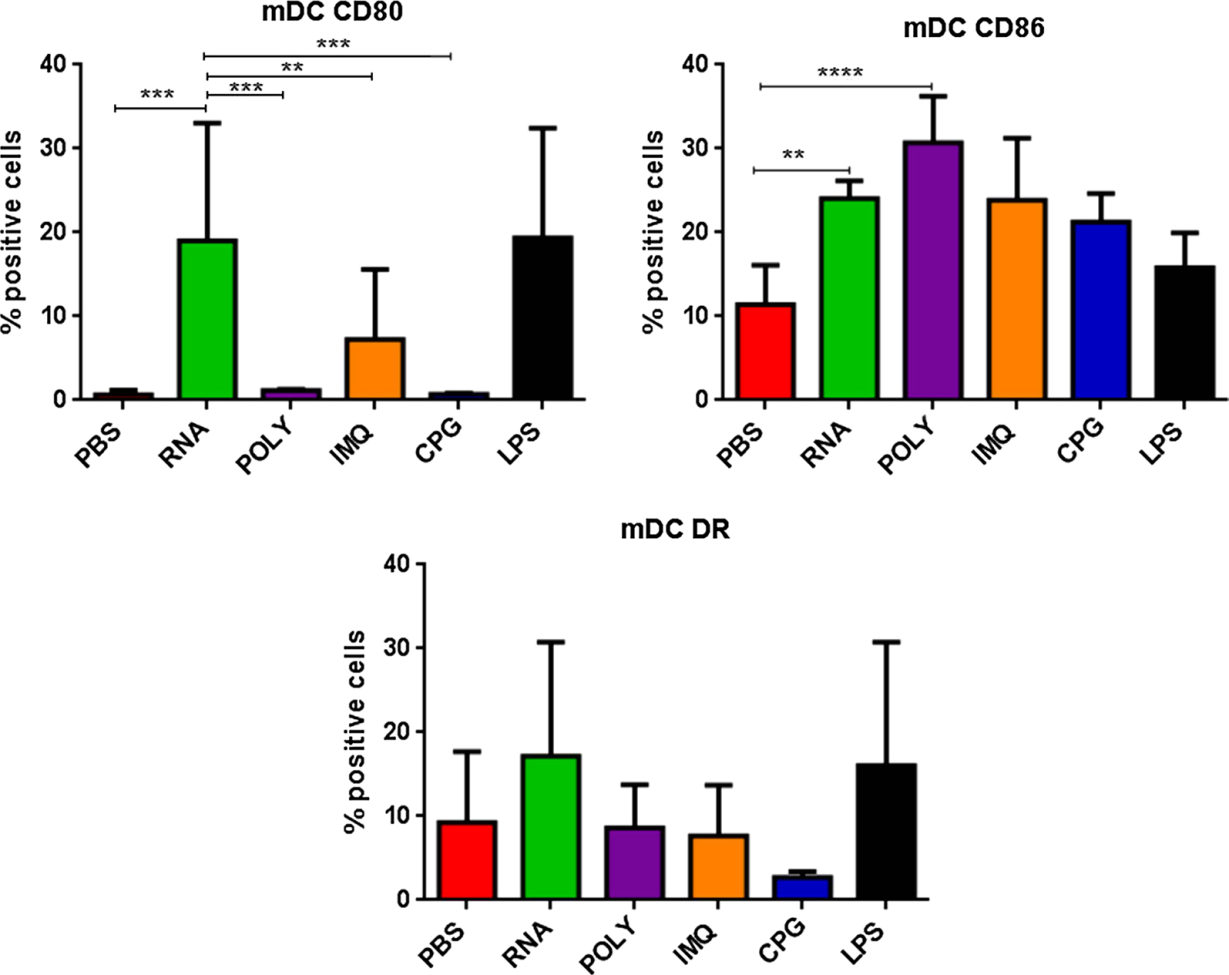
Expression of markers induced by adjuvants in circulating mDCs from healthy subjects. Each of the indicated markers was evaluated by flow cytometry on cells after ex vivo treatment with adjuvants. Results are expressed of percentage of positive cells in the analyzed samples

The same kind of evaluation was performed on CD14^−^CD11c^+^ myeloid dendritic cells (mDCs). Results in OMA patients showed that only the RNAdjuvant^®^ and CpG were able to induce in mDCs a statistical significant increased expression of the CD80 and CD86 maturation markers in samples pre-chemotherapy compared to the negative control, respectively. Moreover, the RNAdjuvant^®^ induced a statistically significant increase of the CD80 molecule in mDCs compared to Poly I:C in pre-treatment samples and CpG in both pre and post-treatment samples. On the contrary, CpG and Poly I:C induced a statistically significant increase of the CD86 molecule in mDCs compared to RNAdjuvant^®^ in both pre and post-chemotherapy samples. No significant difference in expression was observed for the HLA-DR (Fig. 5; Additional file 1: Figure S3). Also for mDCs, patients showed a great variable responsiveness to adjuvants and an example of low and high responders is shown in Fig. 5. Results obtained in mDC derived from OT patients showed a trend to increased expression of the maturation markers compared to the negative control induced by most of the adjuvants analyzed in the study, without reaching a statistical significance. No significant difference was observed between the effects induced by the different adjuvants, with the exception of the CD80, whose expression was significantly induced by the RNAdjuvant^®^ compared to the CpG in post-chemotherapy samples (Fig. 6; Additional file 1: Figure S4). In healthy subjects the effects of adjuvants were evident and statistically significant only for the CD80 and CD86. In particular, the RNAdjuvant^®^ increased the expression of both activation markers compared to negative control. Moreover, the RNAdjuvant^®^ induced the strongest expression of the CD80 compared to all other adjuvants. Finally, the Poly I:C increased the expression of the CD86 marker compared to the negative control (Fig. 7).

### Induction of maturation markers in monocytes by different adjuvants

**Fig. 8:**
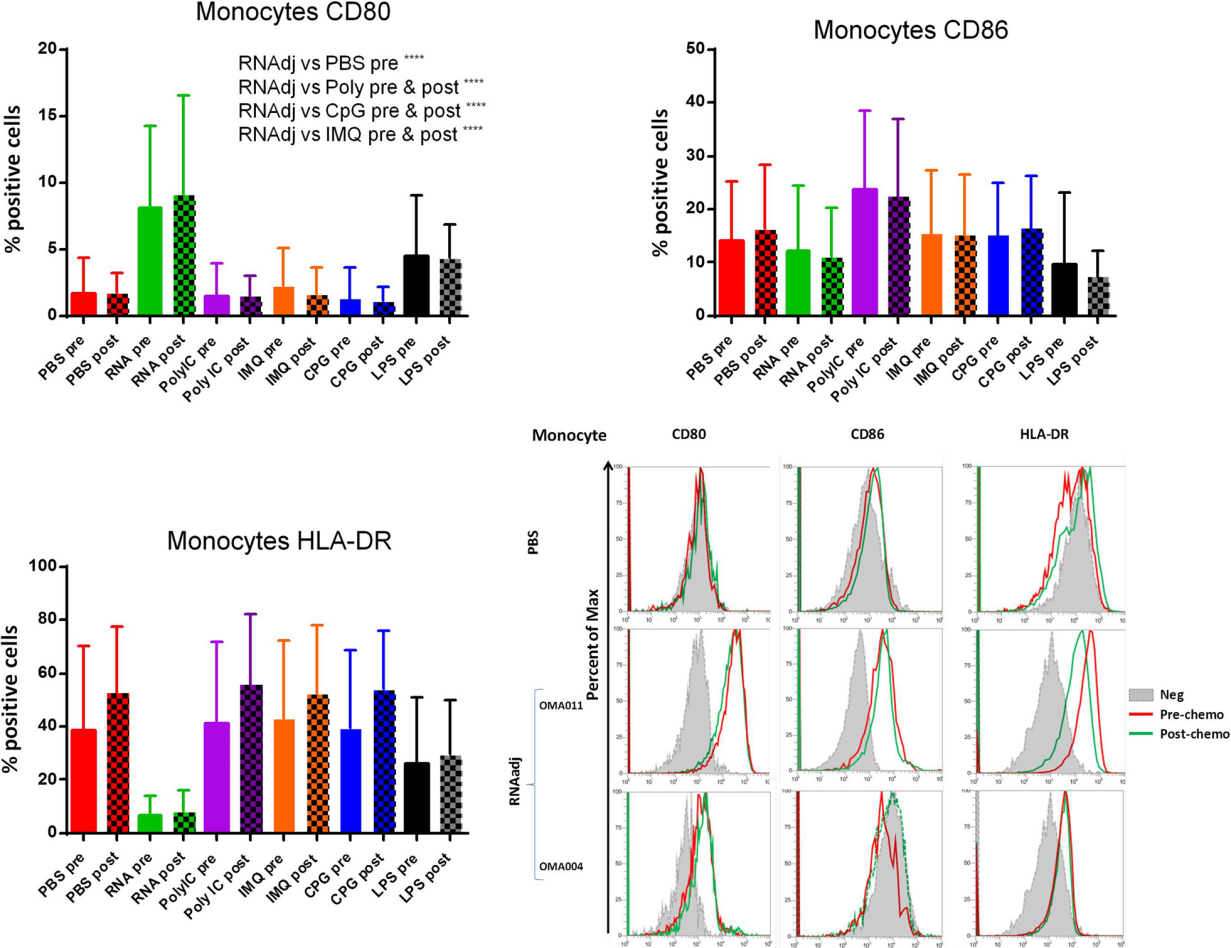
Expression of markers induced by adjuvants in circulating monocytes from colon ca patients. Each of the indicated markers was evaluated by flow cytometry on cells after ex vivo treatment with adjuvants. Samples from colon cancer patients were collected pre and post-chemotherapy. Results are expressed of percentage of positive cells in the analyzed samples. Expression of individual markers induced by RNAdjuvant^®^ in two individual patients is shown as plot

**Fig. 9:**
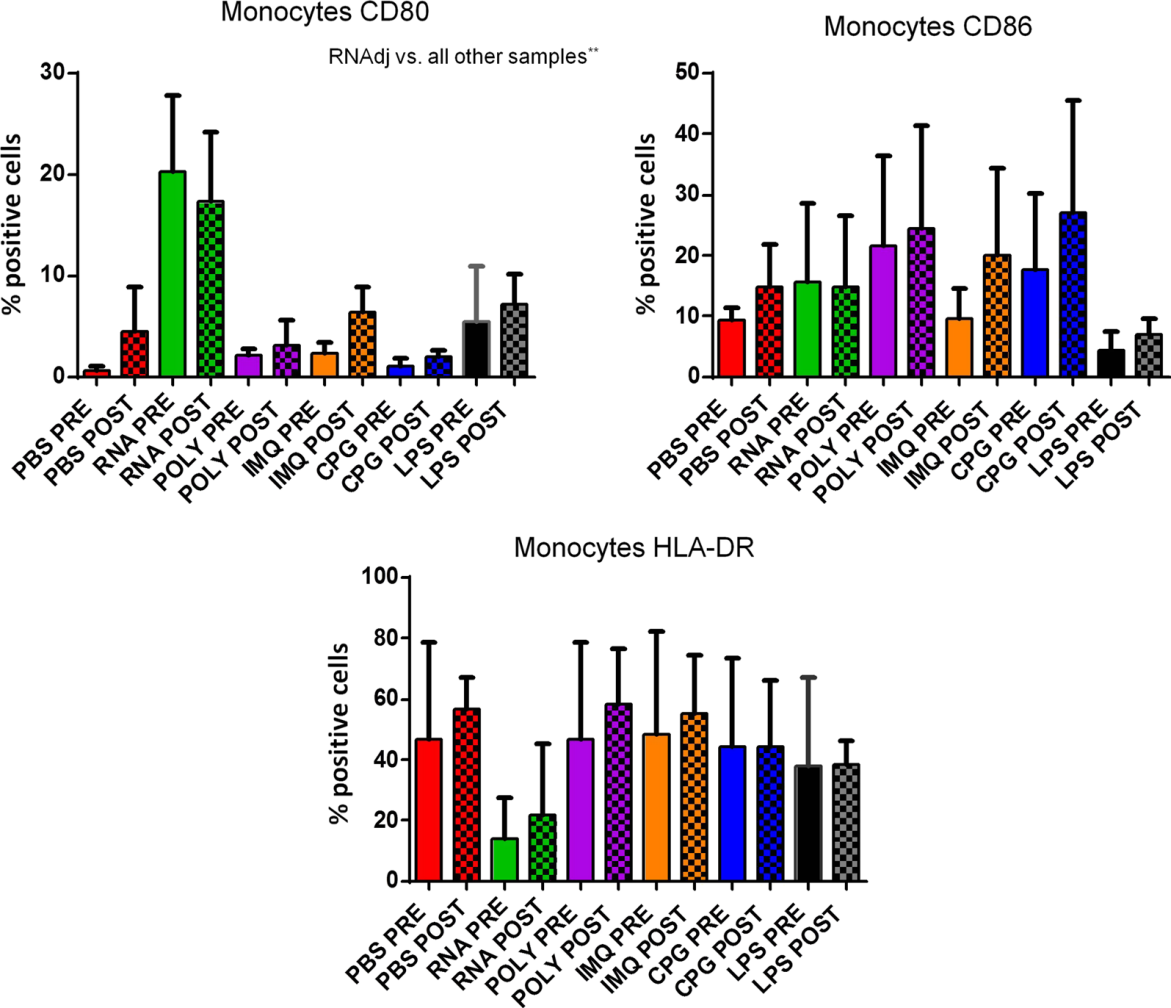
Expression of markers induced by adjuvants in circulating monocytes from lung ca patients. Each of the indicated markers was evaluated by flow cytometry on cells after ex vivo treatment with adjuvants. Samples from lung cancer patients were collected pre and post-chemotherapy. Results are expressed of percentage of positive cells in the analyzed samples

**Fig. 10:**
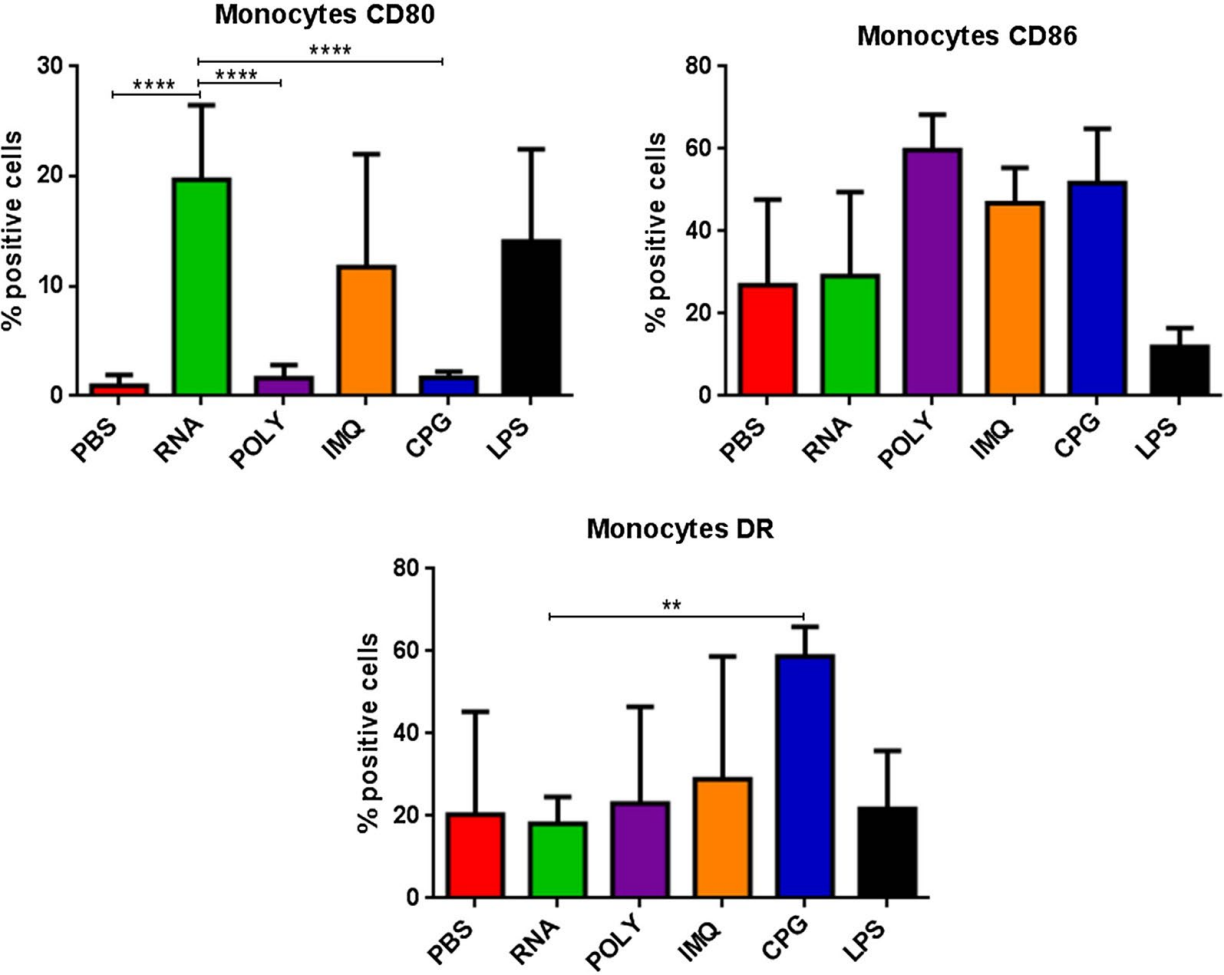
Expression of markers induced by adjuvants in circulating monocytes from healthy subjects. Each of the indicated markers was evaluated by flow cytometry on cells after ex vivo treatment with adjuvants. Results are expressed of percentage of positive cells in the analyzed samples

Finally, the expression of CD80, CD86 and HLA-DR molecules was evaluated in CD14^+^ monocytes. Results in OMA patients showed that only the RNAdjuvant^®^ induced a statistical significant increased expression of the CD80 compared to the negative control in samples pre and post-chemotherapy. Moreover, the RNAdjuvant^®^ induced a statistically significant increase of the CD80 molecule compared to all other adjuvants in pre and post-chemotherapy samples. The expression of CD86 was not significantly increased by adjuvants compared to the negative control, with the exception of Poly I:C in pre-chemotherapy samples. Similar results were observed for HLA-DR, with the exception of a surprising significant down regulation induced by RNAdjuvant^®^ (Fig. 8; Additional file 1: Figure S5). An example of low and high responders to RNAdjuvant^®^ is shown in Fig. 8. Results obtained in monocytes derived from OT patients showed that only the RNAdjuvant^®^ induced an increased expression of CD80 compared to both negative control and other adjuvants in pre and post-chemotherapy samples. The expression of CD86 and HLA-DR was not significantly increased by adjuvants compared to the negative control. As for the OMA samples, the HLA-DR was down regulated by RNAdjuvant^®^ in OT samples without reaching a statistical significance (Fig. 9; Additional file 1: Figure S6). Results obtained in monocytes from healthy subjects were similar to those obtained in pDCs and mDCs of the same subjects (Fig. 10).

### T cell maturation induced by the adjuvants

**Fig. 11:**
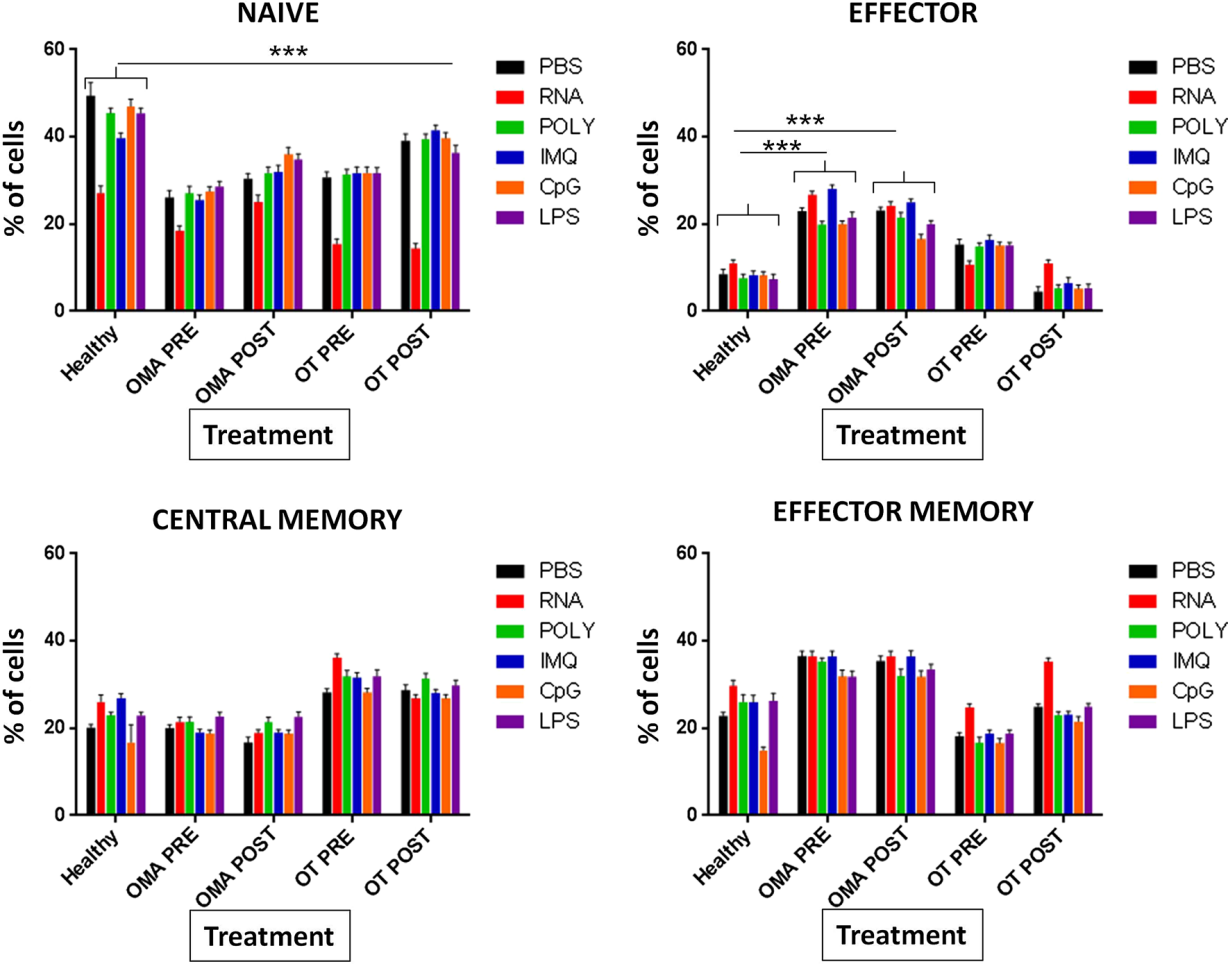
Effect of adjuvants on ex vivo differentiation of naïve CD4^+^ T cells from cancer patients and healthy subjects. Isolated PBMCs were incubated with adjuvants and phenotype of CD4^+^ T cells was assessed by flow cytometry. Naïve (TN) CD4^+^ T were identified as CD4^+^/CD45RA^+^/CD62L^+^; Effector cells (TE) as CD4^+^/CD45RA^+^/CD62L^−^; Central Memory (TCM) as CD4^+^/CD45RO^+^/CD62L^+^; Effector Memory (TEM) as CD4^+^/CD45RO^+^/CD62L^−^. In the panel of Naïve cells, the samples treated with RNAdjuvant are significantly lower compared to samples treated with PBS in Healthy, OT-pre and OT-post subjects (p < 0.01)

The effect on CD4^+^ T cell phenotyping induced by APCs treated with different adjuvants was evaluated in PBMCs 7 days post-treatment ex vivo. Results showed that, at baseline, cancer patients pre and post-chemotherapy had a significantly lower % of naïve T cells (TN) compared to healthy subjects. Treatment with RNAdjuvant^®^ induced a significant further reduction of TN in all subjects, while all other adjuvants did not have a significant effect. Concerning the effector T cells (TE), OMA patients pre and post-chemotherapy showed at baseline a significantly higher percentage of cells compare to healthy subjects. The same pattern was observed in OT patients only pre-chemotherapy, while those post-chemotherapy did not differ from the healthy subject. Treatment with adjuvants did not induce any difference compared to negative control. Central memory T cells (TCM) were slightly increased in OT patients pre and post-chemotherapy compared to healthy subjects without reaching a statistical significance; moreover, treatment with adjuvants did not induce any difference compared to negative control in samples from cancer patients. Effector memory T cells (TEM) were significantly increased in OMA patients pre and post-chemotherapy compared to healthy subjects; also in this case, treatment with adjuvants did not induce any difference compared to negative control in samples from cancer patients. Overall, pre and post-chemotherapy samples did not show any significant difference (Fig. 11).

### Cytokine and chemokines pattern induced in circulating APCs by different adjuvant

**Fig. 12:**
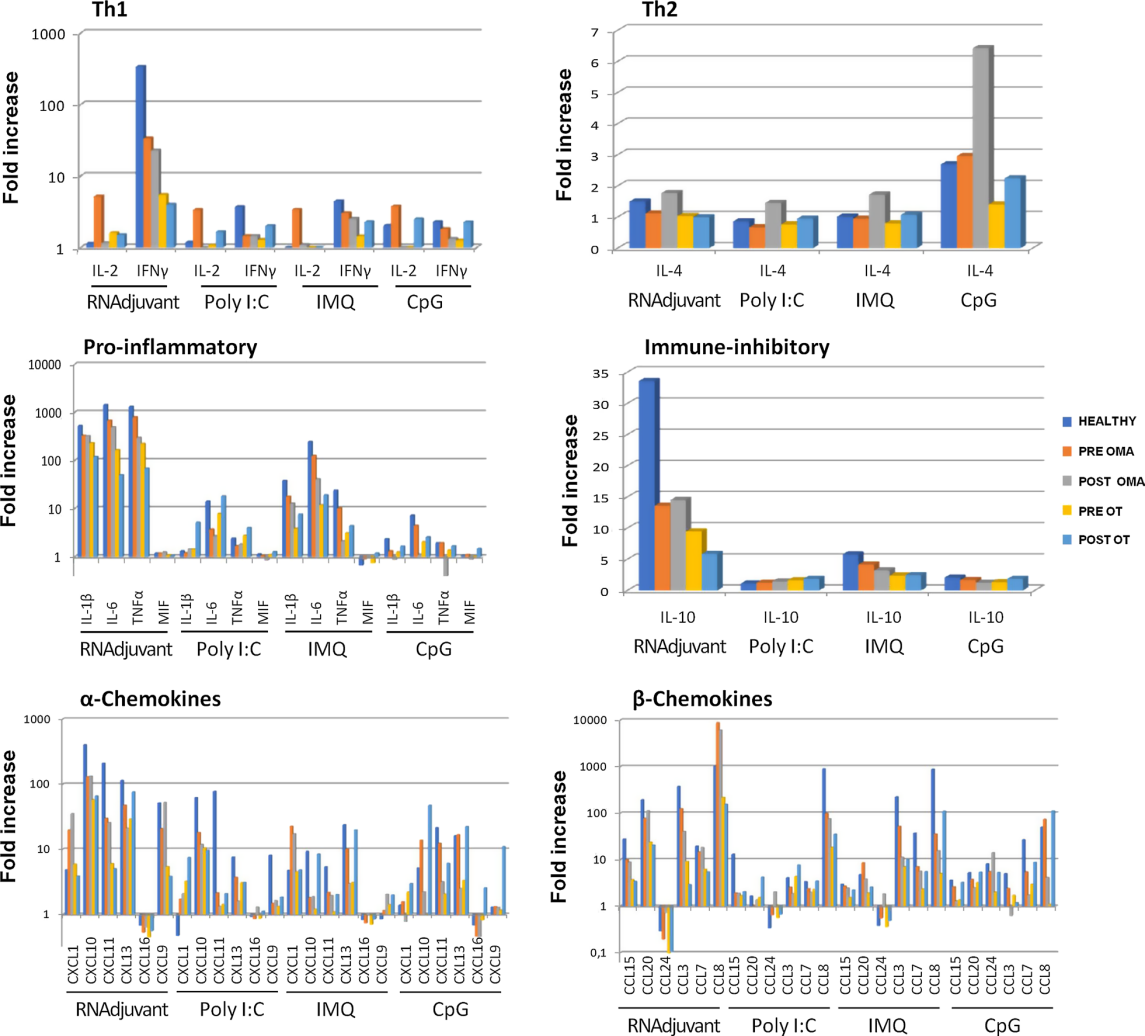
Analysis of cytokine and chemokine production in supernatants of PBMCs from colon (OMA)and lung (OT) cancer patients and healthy subjects. Cytokine and chemokine production was assessed by Bio-Plex Pro Human Chemokine 40-plex Panel (BioRad) in supernatant of PBMCs from colon ca (OMA) and lung ca (OT) patients pre and post chemotherapy, after ex vivo treatment with adjuvants. Bars represent the fold increase calculating the ratio between the mean values (out of a triplicate) of each treatment and the PBS

The level of cytokines Th1 (IL-2 and IFNγ), Th2 (IL-4), pro-inflammatory (IL-6, IL-1Beta, TNF-alpha, MIF) immunosuppressive (IL-10), chemotactic (IL-16) as well as growth factor (GM-CSF), α-chemokines (CX3CL1, CXCL1, CXCL10, CXCL11, CXCL12, CXCL13, CXCL16, CXCL2, CXCL5, CXCL6) and β-chemokines (CCL1 CCL11, CCL13, CCL15, CCL17, CCL19, CCL2, CCL20, CCL21, CCL22, CCL23, CCL24, CCL25, CCL26, CCL27, CCL3, CCL7, CCL8, CXCL9) was assessed by Bio-Plex in supernatants of PBMCs 24 h after treatment with different adjuvants (Fig. 12).

The RNAdjuvant^®^ was the only one to induce high levels of Th1 IFN-γ in cancer patients with no significant difference in pre and post-chemotherapy samples, although at lower level compared to healthy subjects. Pro-inflammatory cytokines were induced in cancer patients mostly by RNAdjuvant^®^ and IMQ, at levels comparable to healthy subjects, but with lower potency in post-chemotherapy samples. None of the adjuvants induced the Th2 IL-4 cytokine, with highest concentrations of 6 pg/ml induced by CpG; moreover, only RNAdjuvant^®^ induced a modest level of the immune-inhibitory IL-10 cytokine. Regarding α and β chemokines, all the adjuvants induced their expression with different potency, although the RNAdjuvant^®^ scored always as the most potent (Fig. 12; Additional file 1: Figures S7 and S8). In most cases, no significant difference was observed between healthy subjects and cancer patients, as well as between pre and post-chemotherapy cancer patients. Chemokines with highest expression were CXCL10, CXCL11 and CXCL13 as well as CCL20, CCL3 and CCL8. Consistently, CXCL16 and CCL24 were down-regulated by all adjuvants.

## Discussion

Vaccines based on peptides show a limited efficiency in eliciting immune responses and require formulations with adjuvants to potentiate their immunogenicity. However, most of the adjuvants are developed for eliciting a Th2-type response by preventive vaccines in healthy subjects. On the contrary, therapeutic cancer vaccines aim to eliciting a Th1-type response in patients affected by cancer and very few adjuvants are available for this purpose. The immunological responsiveness to such adjuvants in cancer patients undergoing chemotherapy has not been previously assessed. In the present study we aimed to verify the level of responsiveness to the adjuvants mostly used in therapeutic cancer vaccine trials, comparing patients affected by colon cancer and non-small cell lung carcinoma, pre and post-chemotherapy, to healthy subjects.

This study was carried out using a multiparametric analysis in an ex vivo setting, treating PBMCs from lung and colon cancer patients as well as from healthy controls with RNAdjuvant^®^, Poly I:C, IMQ and CpG. The results showed that the basal expression of the CD80, CD86 and HLA-DR molecules in monocytes, myeloid dendritic cells (mDCs) and plasmacytoid dendritic cells (pDCs) was largely comparable between healthy subjects and cancer patients, before and after the chemotherapy. However, the basal expression of CD80 and HLA-DR showed a trend of increase after the chemotherapy, possibly suggesting the positive effect of cisplatin on immune cell differentiation and antigen presentation [39–41].

The effects of the adjuvants on pDCs were very limited in cancer patients, pre and post-chemotherapy, compared to healthy subjects. Only the RNAdjuvant^®^ induced a statistically significant increased expression of CD80 and HLA-DR in OT patients compared to the negative control, in pre and post-treatment samples respectively. The effects of adjuvants on mDCs in pre-chemotherapy samples from OMA patients showed that RNAdjuvant^®^ and CpG were able to induce a statistical significant activation of the CD80 and CD86 maturation markers compared to negative samples, respectively. On the contrary, effects on samples from OT patients were not statistically significant. The RNAdjuvant^®^ induced the strongest activation of CD80 expression in monocytes from cancer patients, pre and post-chemotherapy, compared to negative control and other adjuvants. Poly I:C was the only adjuvant to induce an increased expression of CD86 compared to negative control in monocytes from OMA patients pre-chemotherapy. Overall, the results on activation markers showed that cancer patients are still responsive to adjuvants, each of them used in the present study at the individual optimal concentration. A degree of potency was observed, but the RNAdjuvant^®^ scored as the most effective adjuvant. Interestingly, cell subtypes analyzed in the study have been shown to have different expression levels for the individual Toll Like Receptors and should be responsive only to the specific ligands. Indeed, mDCs have high expression of TLR3 as well as relative expression of TLRs7 and 8, but no expression of TLR9, and should respond to all adjuvants tested in the study with exception of CpG [42]. On the contrary, pDCs have high expression only of TLR7 and 9 and should respond to all adjuvants tested with exception of Poly I:C [42]. Monocytes, have low expression of TLRs7, 8 and 9 and should moderately respond to all adjuvants tested with exception of Poly I:C. However, if pDCs are present in cell culture, monocytes have been shown to respond also to the TLR9 agonist CpG [43]. According to the latter evidence, it could be predicted that in a context of mixed population (such as PBMCs) the individual subtypes may respond to TLR agonists even when the target TLRs show low expression. Data obtained after chemotherapy protocols including cisplatin or analogues suggested a positive effect on immune cell differentiation and antigen presentation, as observed in the baseline expression of the markers, although the responsiveness to adjuvants appeared to be reduced. The biological mechanisms of such apparent discrepancy need to be further evaluated by increasing the number of samples analyzed.

The ability of APCs treated with adjuvants to induce activation of naïve CD4^+^ T cells into effector cells was assessed ex vivo. The results showed that, at baseline, pre and post-chemotherapy cancer patients have a significantly lower percentage of naïve T cells (TN) compared to healthy subjects. This would suggest that the pathological condition, regardless the chemotherapy, may have an impact on the fraction of T cells available to respond to new antigens. Such a reduction was further induced by treatment with RNAdjuvant^®^, while no effect was observed upon treatment with the other adjuvants. Interestingly, colon and lung cancer patients showed a different pattern for the other phenotypes. Indeed, at baseline, while OMA samples showed a significantly higher percentage of TE and TEM compared to healthy subjects, OT patients showed a higher percentage of TCM compared to healthy subjects. For all the latter phenotypes, treatment with adjuvants did not induce a significant change in the percentage compared to the negative control. Overall, no statistical deviation from data in healthy subjects was observed in cancer patients and no difference was observed between samples from cancer patients pre and post-chemotherapy. This is of high relevance, indicating that the pool of effector and memory CD4^+^ T cells is fully preserved in cancer patients. In particular, effector memory (TEM) and central memory (TCM) cells are capable of circulating in lymphoid (TCM) as well as non-lymphoid compartments (TEM) [44, 45]. Therefore, upon contact with the appropriate antigen, effector memory cells can execute effector functions instantly, whereas central or lymphoid memory cells can rapidly proliferate, expanding and acquiring effector functions.

Among the adjuvants evaluated in the present study, RNAdjuvant^®^ was the only one to induce high levels of Th1 IFN-γ in cancer patients with no significant difference in pre and post-chemotherapy samples. On the contrary, none of the adjuvants induced IL-4 which is considered one the major Th2 cytokines. Such results suggest that RNAdjuvant^®^ represents the most potent Th1 inducer to be combined in a therapeutic cancer vaccine formulation. The same adjuvant is also the most potent inducer of pro-inflammatory cytokines as well as of α and β chemokines, suggesting a great capability of initiating the innate immune response coupled to the ability of recruiting immune cells, especially T cells, for potentiating the effector arm of anti-cancer immunity. Interestingly, the biological meaning of the down-expression of CXCL16 and CCL24 induced by all adjuvants needs to be further investigated in a larger experimental setting. In most cases, although at lower level compared to healthy subjects, cytokine level in cancer patients was significantly high and no statistical difference was observed between pre and post-chemotherapy samples. In some cases, OMA patients showed a higher level of cytokine production compared to OT patients. Such an observation needs to be confirmed on a larger group of patients.

## Conclusion

The immunomodulatory profile of the RNAdjuvant^®^ appears to be potent and complete compared to the other adjuvants widely used in cancer vaccine clinical trials. Moreover, our results showed that most of the effects in cancer patients, although often of lower potency compared to healthy subjects, are suggestive of a valuable immune responsiveness. Furthermore, the limited differences observed between pre and post-chemotherapy samples indicated that cancer patients may well respond to therapeutic cancer vaccines even after chemotherapy.

## Supplementary information

**Additional file 1: Figure S1.** Expression of markers induced by adjuvants in circulating pDCs from colon ca patients. Each of the indicated markers was evaluated by flow cytometry on cells after ex vivo treatment with adjuvants. Samples from colon cancer patients were collected pre and post-chemotherapy. Results are expressed of percentage of positive cells in the analyzed samples. **Figure S2.** Expression of markers induced by adjuvants in circulating pDCs from lung ca patients. Each of the indicated markers was evaluated by flow cytometry on cells after ex vivo treatment with adjuvants. Samples from lung cancer patients were collected pre and post-chemotherapy. Results are expressed of percentage of positive cells in the analyzed samples. **Figure S3.** Expression of markers induced by adjuvants in circulating mDCs from colon ca patients. Each of the indicated markers was evaluated by flow cytometry on cells after ex vivo treatment with adjuvants. Samples from colon cancer patients were collected pre and post-chemotherapy. Results are expressed of percentage of positive cells in the analyzed samples. **Figure S4.** Expression of markers induced by adjuvants in circulating mDCs from lung ca patients. Each of the indicated markers was evaluated by flow cytometry on cells after ex vivo treatment with adjuvants. Samples from lung cancer patients were collected pre and post-chemotherapy. Results are expressed of percentage of positive cells in the analyzed samples. **Figure S5.** Expression of markers induced by adjuvants in circulating monocytes from colon ca patients. Each of the indicated markers was evaluated by flow cytometry on cells after ex vivo treatment with adjuvants. Samples from colon cancer patients were collected pre and post-chemotherapy. Results are expressed of percentage of positive cells in the analyzed samples. **Figure S6.** Expression of markers induced by adjuvants in circulating monocytes from lung ca patients. Each of the indicated markers was evaluated by flow cytometry on cells after ex vivo treatment with adjuvants. Samples from lung cancer patients were collected pre and post-chemotherapy. Results are expressed of percentage of positive cells in the analyzed samples. **Figure S7.** Analysis of alpha chemokine production in supernatants of PBMCs from cancer patients and healthy subjects. Cytokine and chemokine production was assessed by Bio-Plex Pro Human Chemokine 40-plex Panel (BioRad) in supernatant of PBMCs treated ex vivo with adjuvants. **Figure S8.** Analysis of beta chemokine production in supernatants of PBMCs from cancer patients and healthy subjects. Cytokine and chemokine production was assessed by Bio-Plex Pro Human Chemokine 40-plex Panel (BioRad) in supernatant of PBMCs treated ex vivo with adjuvants.

## Data Availability

Data and material are available upon request.

## Abbreviations

APC: antigen-presenting cell
CCL-n: chemokine (C-C motif) ligands
CTL: cytotoxic T lymphocytes
CXCL-n: chemokine (C-X-C motif) ligands
GM-CSF: Granulocyte-Macrophage Colony-Stimulating Factor
HCC: hepatocellular carcinoma
IFN-γ: interferon gamma
IL-n: interleukins
IMQ: imiquimod
LPS: lipopolysaccharide
mDC: myeloid dendritic cell
MIF: migration inhibitory factor
OMA: colon cancer patients
OT: lung cancer patients
PAMPs: pathogen-associated molecular patterns
PBMCs: peripheral blood mononuclear cells
PBS: phosphate-buffered saline
pDC: plasmacytoid dendritic cell
Poly I:C: polyinosinic–polycytidylic acid
PRRs: pattern recognition receptors
RIG-I: retinoic acid-inducible gene 1-like receptor
TCM: central memory T cell
TE: effector T cell
TEM: effector memory T cell
Th1: T helper 1
Th2: T helper 2
TLRs: toll-like receptors
TN: Naïve T cells
TNF-alpha: tumor necrosis factor α
