# Supplementary material for: Immunological effects of adjuvants in subsets of antigen presenting cells of cancer patients undergoing chemotherapy
Source: J Transl Med. 2020 Jan 23;18:34. doi: 10.1186/s12967-020-02218-x (PMC6977281; doi:10.1186/s12967-020-02218-x)
Supplement: Supplementary file 1 — Additional file 1: Figure S1. Expression of markers induced by adjuvants in circulating pDCs from colon ca patients. Each of the indicated markers was evaluated by flow cytometry on cells after ex vivo treatment with adjuvants. Samples from colon cancer patients were collected pre and post-chemotherapy. Results are expressed of percentage of positive cells in the analyzed samples. Figure S2. Expression of markers induced by adjuvants in circulating pDCs from lung ca patients. Each of the indicated markers was evaluated by flow cytometry on cells after ex vivo treatment with adjuvants. Samples from lung cancer patients were collected pre and post-chemotherapy. Results are expressed of percentage of positive cells in the analyzed samples. Figure S3. Expression of markers induced by adjuvants in circulating mDCs from colon ca patients. Each of the indicated markers was evaluated by flow cytometry on cells after ex vivo treatment with adjuvants. Samples from colon cancer patients were collected pre and post-chemotherapy. Results are expressed of percentage of positive cells in the analyzed samples. Figure S4. Expression of markers induced by adjuvants in circulating mDCs from lung ca patients. Each of the indicated markers was evaluated by flow cytometry on cells after ex vivo treatment with adjuvants. Samples from lung cancer patients were collected pre and post-chemotherapy. Results are expressed of percentage of positive cells in the analyzed samples. Figure S5. Expression of markers induced by adjuvants in circulating monocytes from colon ca patients. Each of the indicated markers was evaluated by flow cytometry on cells after ex vivo treatment with adjuvants. Samples from colon cancer patients were collected pre and post-chemotherapy. Results are expressed of percentage of positive cells in the analyzed samples. Figure S6. Expression of markers induced by adjuvants in circulating monocytes from lung ca patients. Each of the indicated markers was evaluated by flow c [file 12967_2020_2218_MOESM1_ESM.pptx]

## Slide 1
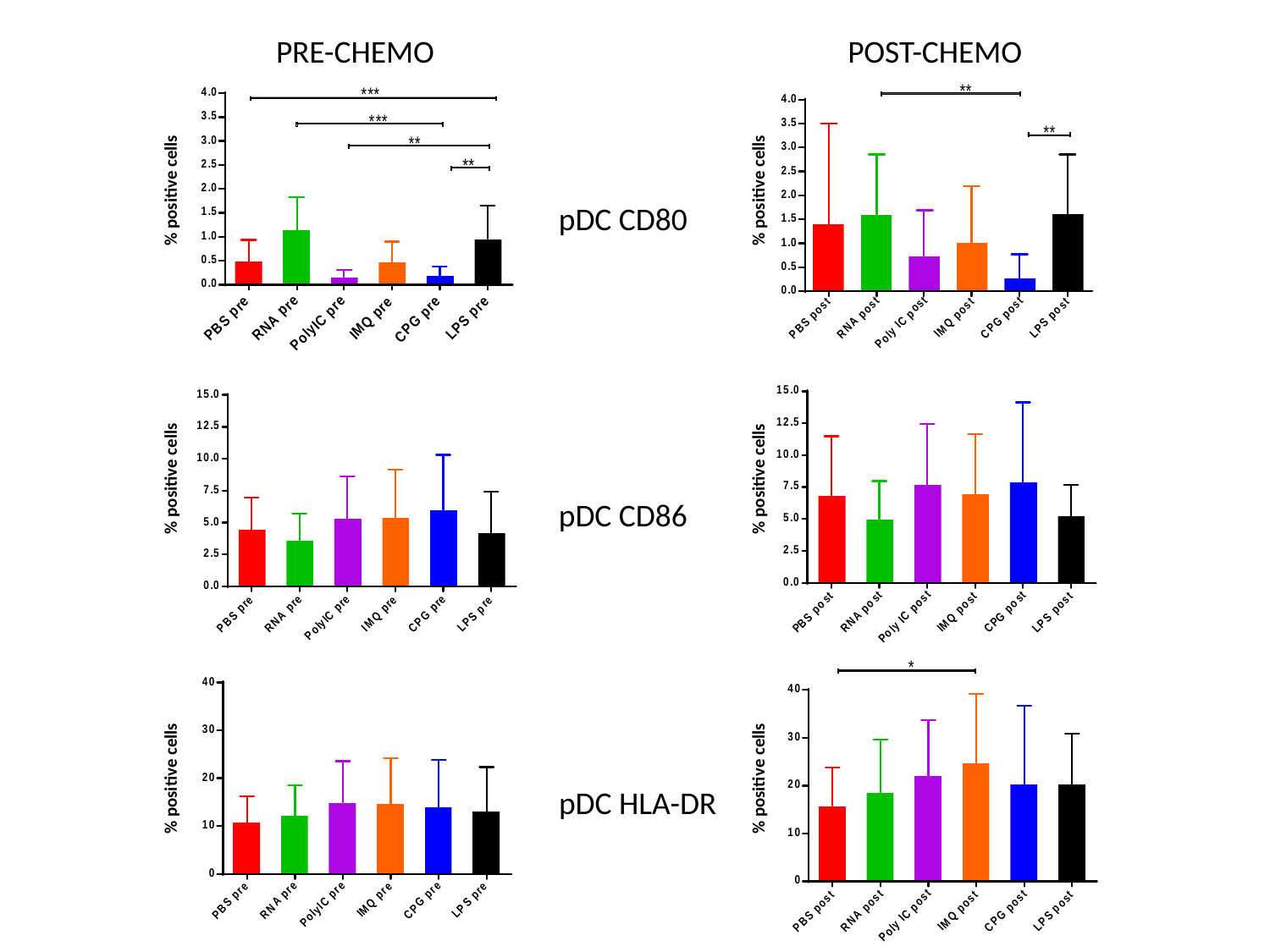

PRE-CHEMO
POST-CHEMO
% positive cells
% positive cells
pDC CD80
% positive cells
% positive cells
pDC CD86
% positive cells
% positive cells
pDC HLA-DR

## Slide 2
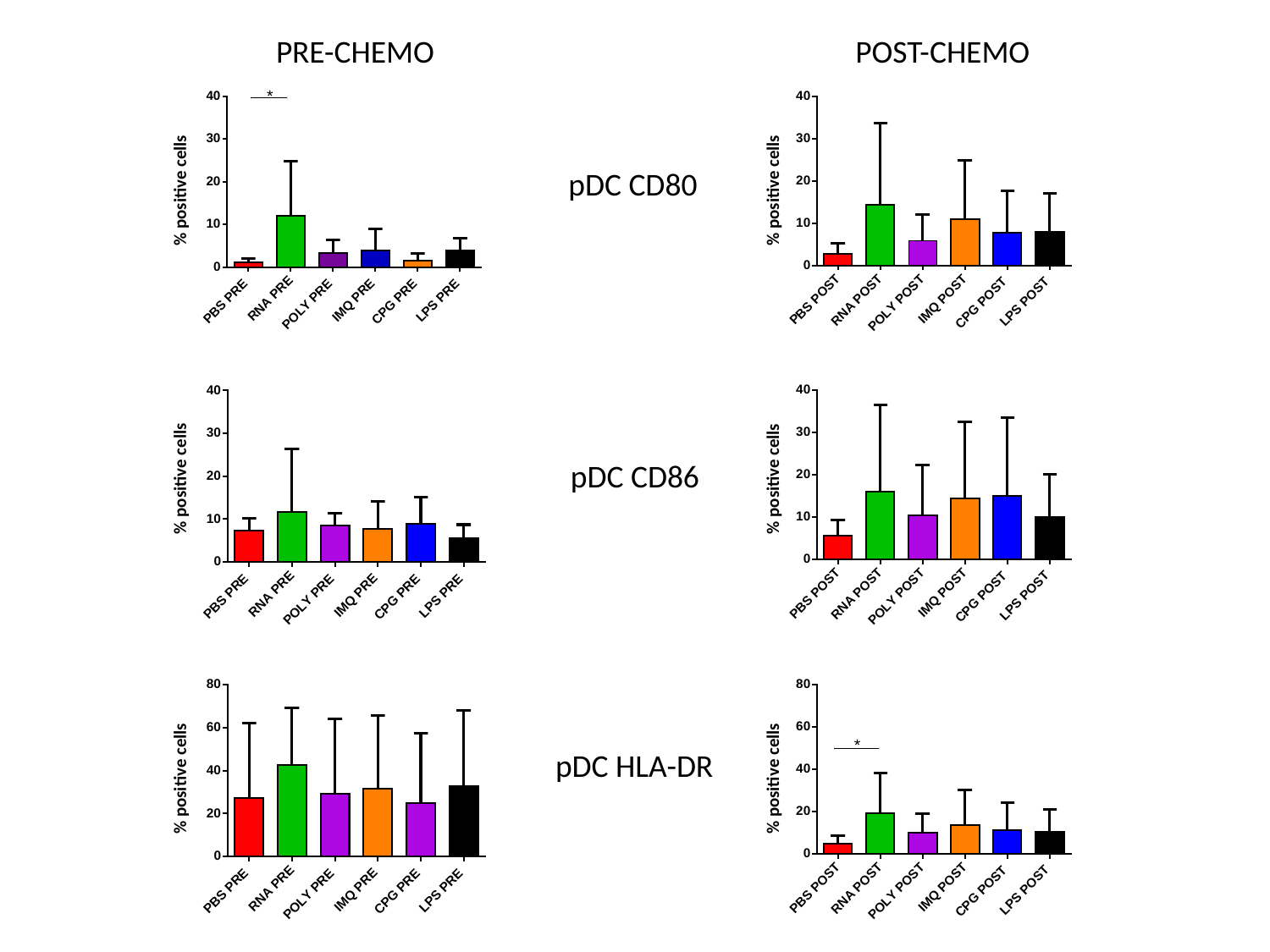

PRE-CHEMO
POST-CHEMO
pDC CD80
% positive cells
% positive cells
pDC CD86
% positive cells
% positive cells
pDC HLA-DR
% positive cells
% positive cells

## Slide 3
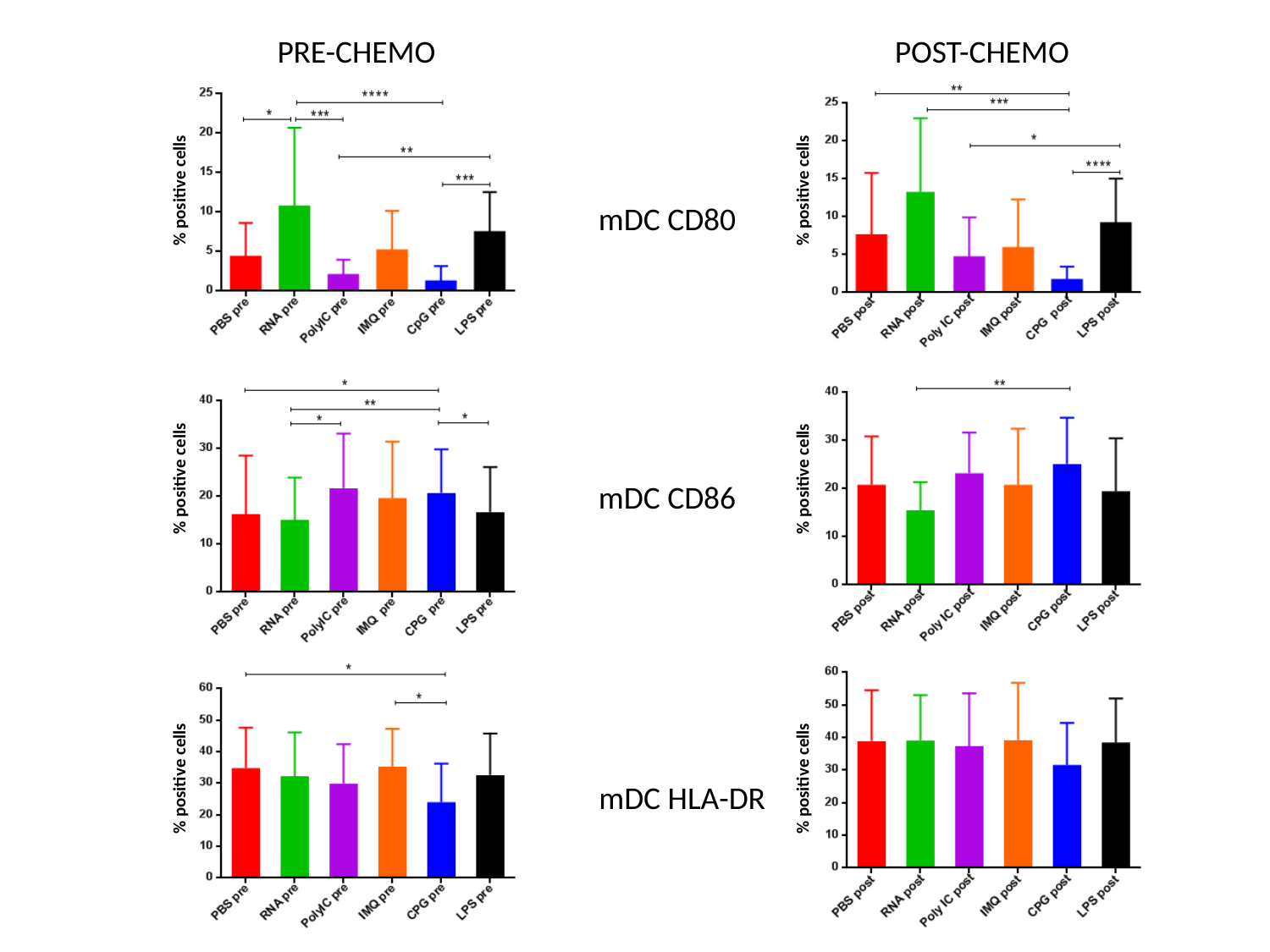

PRE-CHEMO
POST-CHEMO
% positive cells
% positive cells
mDC CD80
% positive cells
% positive cells
mDC CD86
% positive cells
% positive cells
mDC HLA-DR

## Slide 4
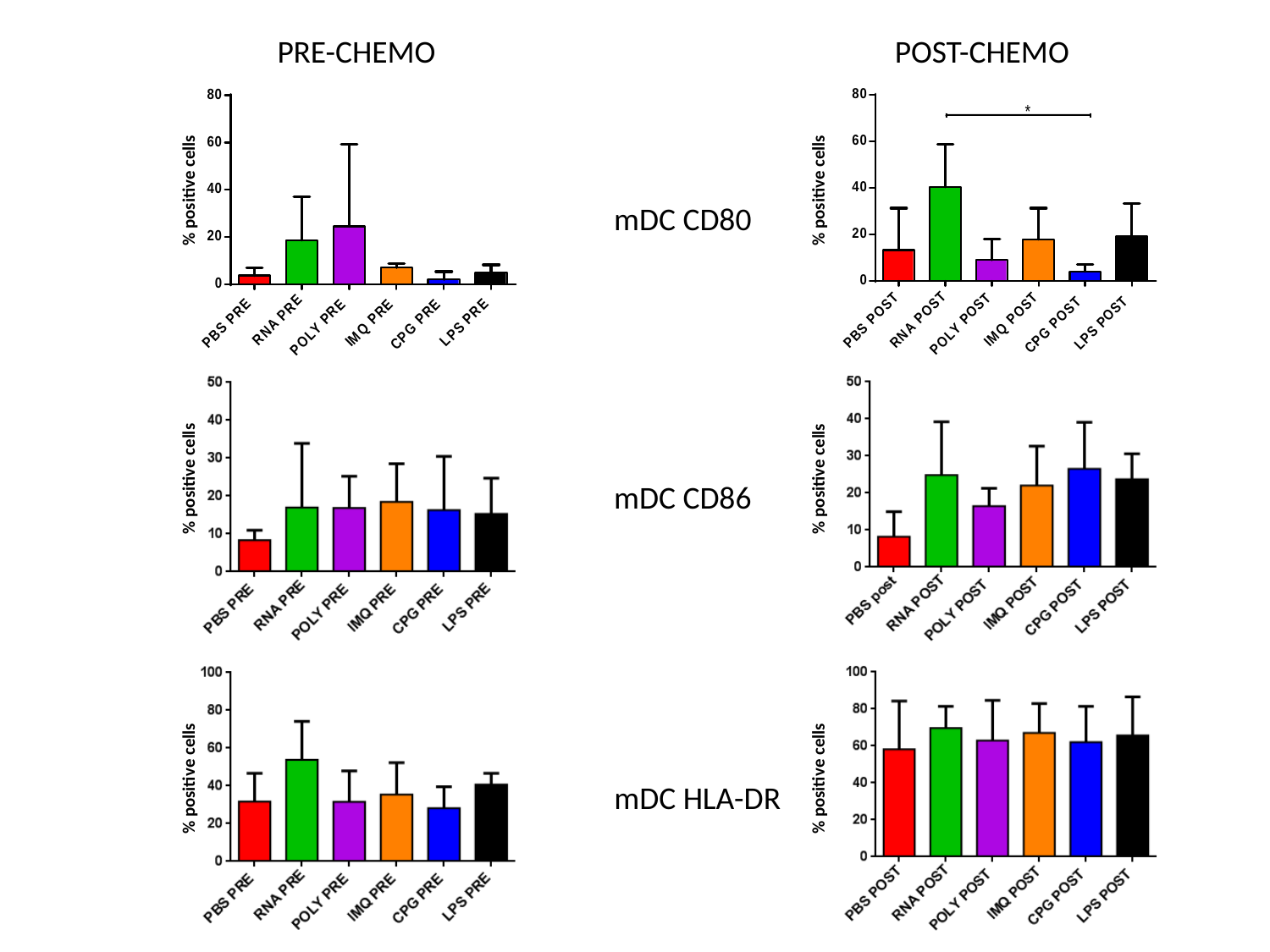

PRE-CHEMO
POST-CHEMO
% positive cells
% positive cells
mDC CD80
% positive cells
% positive cells
mDC CD86
% positive cells
% positive cells
mDC HLA-DR

## Slide 5
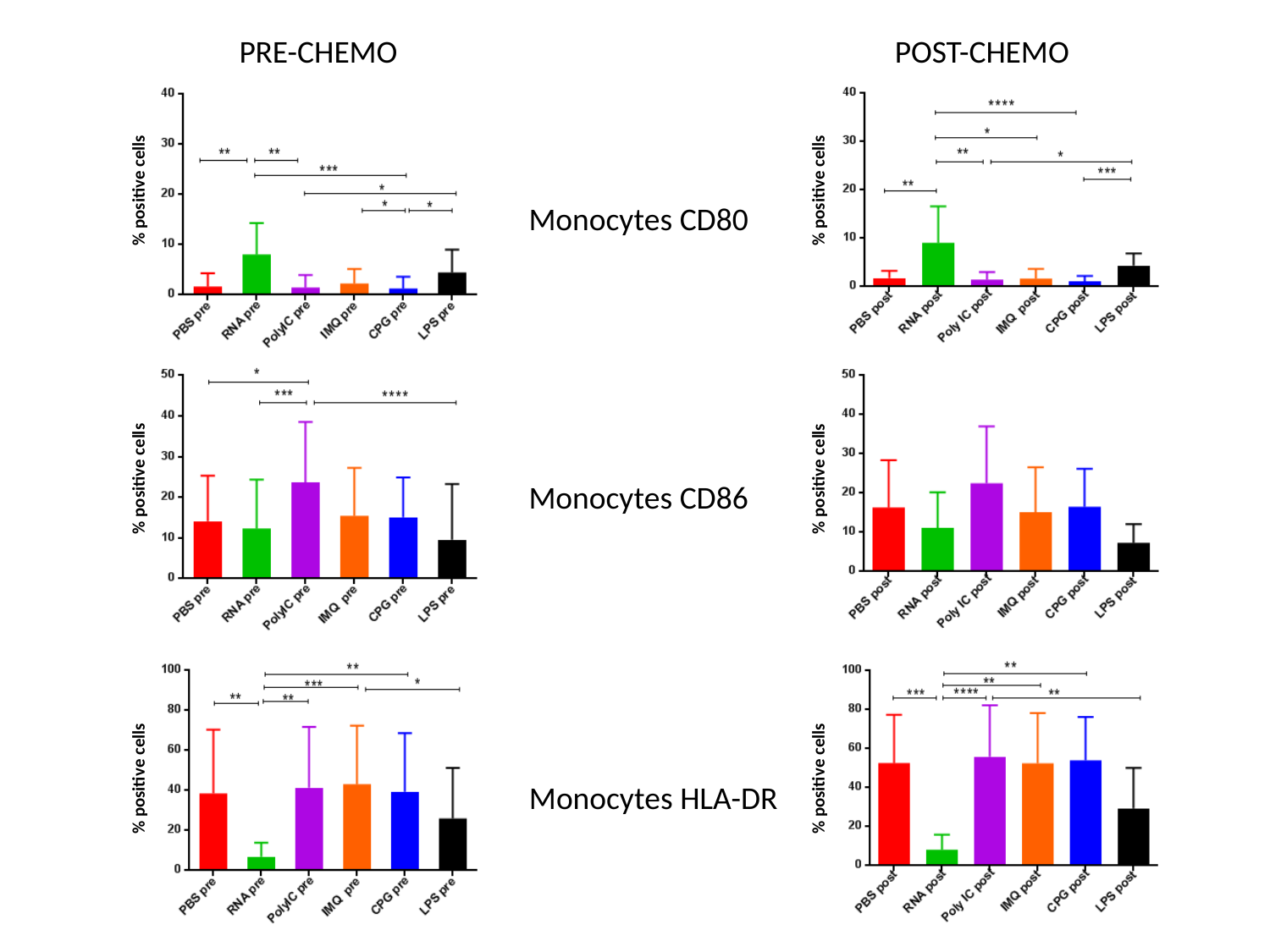

PRE-CHEMO
POST-CHEMO
% positive cells
% positive cells
Monocytes CD80
% positive cells
% positive cells
Monocytes CD86
% positive cells
% positive cells
Monocytes HLA-DR

## Slide 6
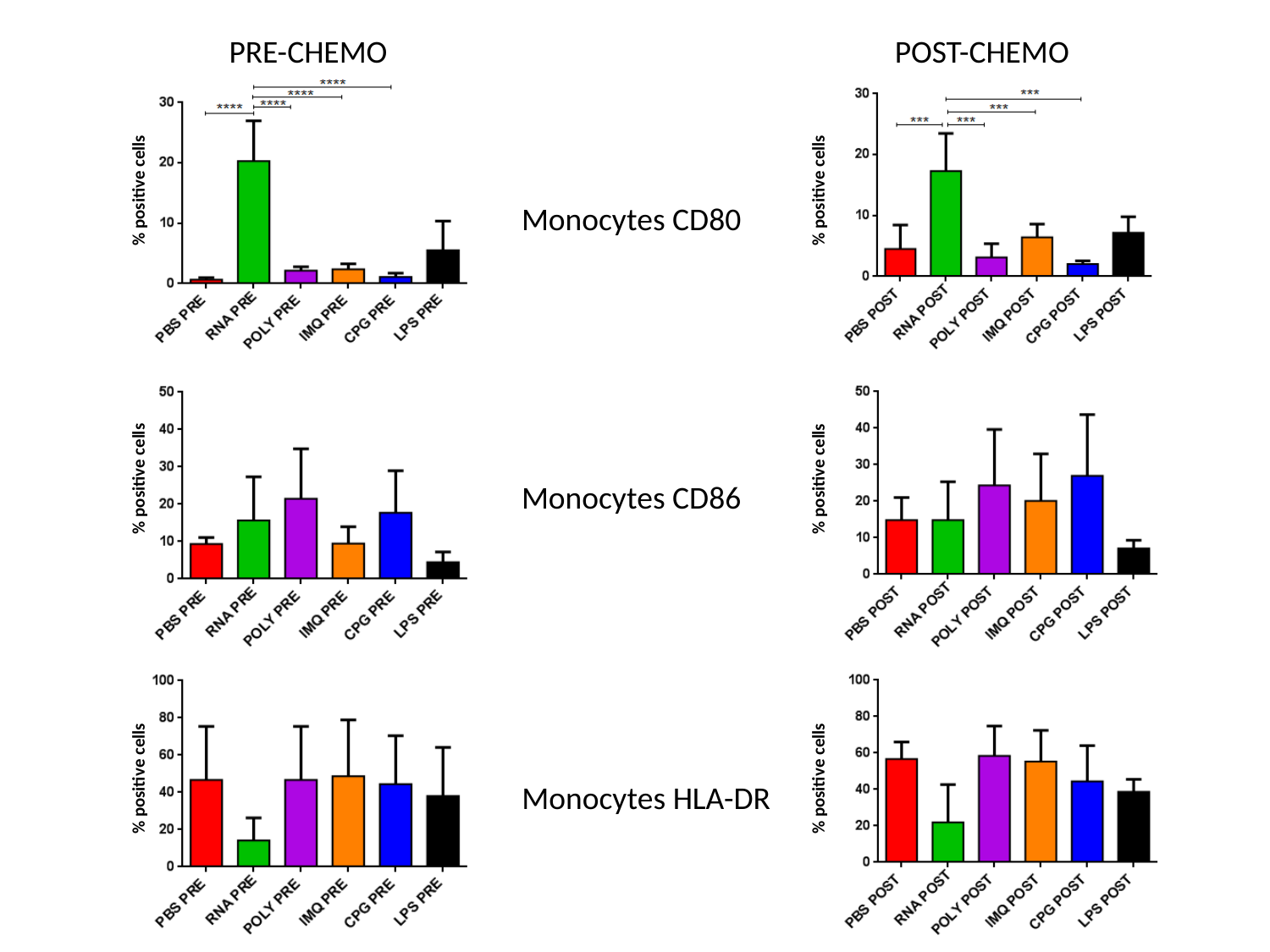

PRE-CHEMO
POST-CHEMO
% positive cells
% positive cells
Monocytes CD80
% positive cells
% positive cells
Monocytes CD86
% positive cells
% positive cells
Monocytes HLA-DR

## Slide 7
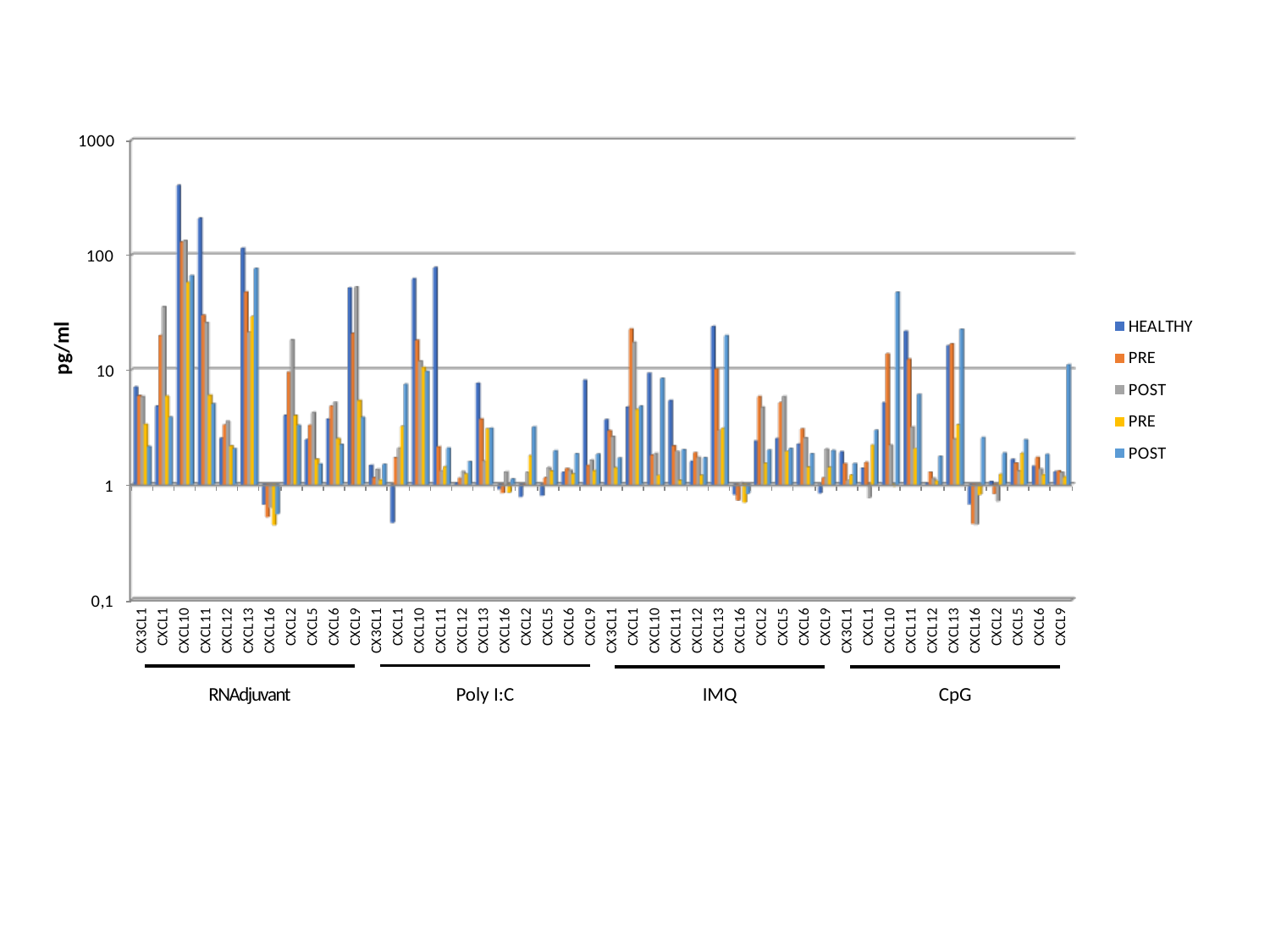

pg/ml
RNAdjuvant
Poly I:C
IMQ
CpG

## Slide 8
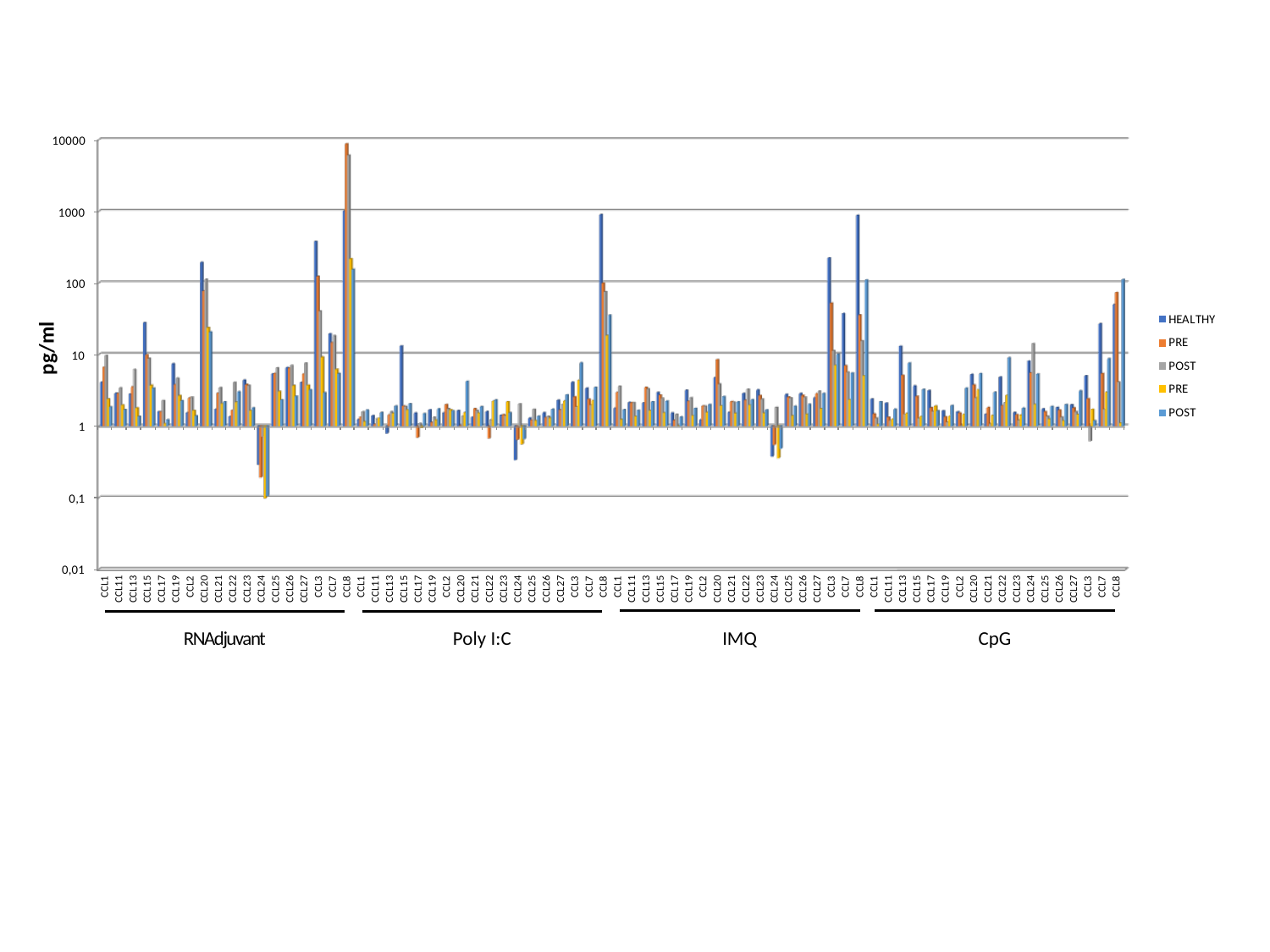

pg/ml
RNAdjuvant
Poly I:C
IMQ
CpG
